# Cancer cell-specific and pro-apoptotic SMAC peptide-doxorubicin conjugated prodrug encapsulated aposomes for synergistic cancer immunotherapy

**DOI:** 10.1186/s12951-024-02314-w

**Published:** 2024-03-13

**Authors:** Jinseong Kim, Man Kyu Shim, Yujeong Moon, Jeongrae Kim, Hanhee Cho, Wan Su Yun, Nayeon Shim, Joon-Kyung Seong, Yonghyun Lee, Dong-Kwon Lim, Kwangmeyung Kim

**Affiliations:** 1 College of Pharmacy, Graduate School of Pharmaceutical Sciences, Ewha Womans University, Seoul, 03760 Republic of Korea; 2 KU-KIST Graduate School of Converging Science and Technology, Korea University, Seoul, 02841 Republic of Korea; 3 Medicinal Materials Research Center, Biomedical Research Division, Korea Institute of Science and Technology (KIST), Seoul, 02792 Republic of Korea; 4 Department of Bioengineering, Korea University, Seoul, 02841 Republic of Korea

**Keywords:** Aposomes, Cancer immunotherapy, Immune checkpoint blockade, PEGylated liposome, Immunogenic cell death, Drug resistance

## Abstract

**Background:**

Immunogenic cell death (ICD) is a crucial approach to turn immunosuppressive tumor microenvironment (ITM) into immune-responsive milieu and improve the response rate of immune checkpoint blockade (ICB) therapy. However, cancer cells show resistance to ICD-inducing chemotherapeutic drugs, and non-specific toxicity of those drugs against immune cells reduce the immunotherapy efficiency.

**Methods:**

Herein, we propose cancer cell-specific and pro-apoptotic liposomes (Aposomes) encapsulating second mitochondria-derived activator of caspases mimetic peptide (SMAC-P)-doxorubicin (DOX) conjugated prodrug to potentiate combinational ICB therapy with ICD. The SMAC-P (*AVPIAQ*) with cathepsin B-cleavable peptide (*FRRG*) was directly conjugated to DOX, and the resulting SMAC-P-FRRG-DOX prodrug was encapsulated into PEGylated liposomes.

**Results:**

The SMAC-P-FRRG-DOX encapsulated PEGylated liposomes (Aposomes) form a stable nanostructure with an average diameter of 109.1 ± 5.14 nm and promote the apoptotic cell death mainly in cathepsin B-overexpressed cancer cells. Therefore, Aposomes induce a potent ICD in targeted cancer cells in synergy of SMAC-P with DOX in cultured cells. In colon tumor models, Aposomes efficiently accumulate in targeted tumor tissues via enhanced permeability and retention (EPR) effect and release the encapsulated prodrug of SMAC-P-FRRG-DOX, which is subsequently cleaved to SMAC-P and DOX in cancer cells. Importantly, the synergistic activity of inhibitors of apoptosis proteins (IAPs)-inhibitory SMAC-P sensitizing the effects of DOX induces a potent ICD in the cancer cells to promote dendritic cell (DC) maturation and stimulate T cell proliferation and activation, turning ITM into immune-responsive milieu.

**Conclusions:**

Eventually, the combination of Aposomes with anti-PD-L1 antibody results in a high rate of complete tumor regression (CR: 80%) and also prevent the tumor recurrence by immunological memory established during treatments.

**Graphical Abstract:**

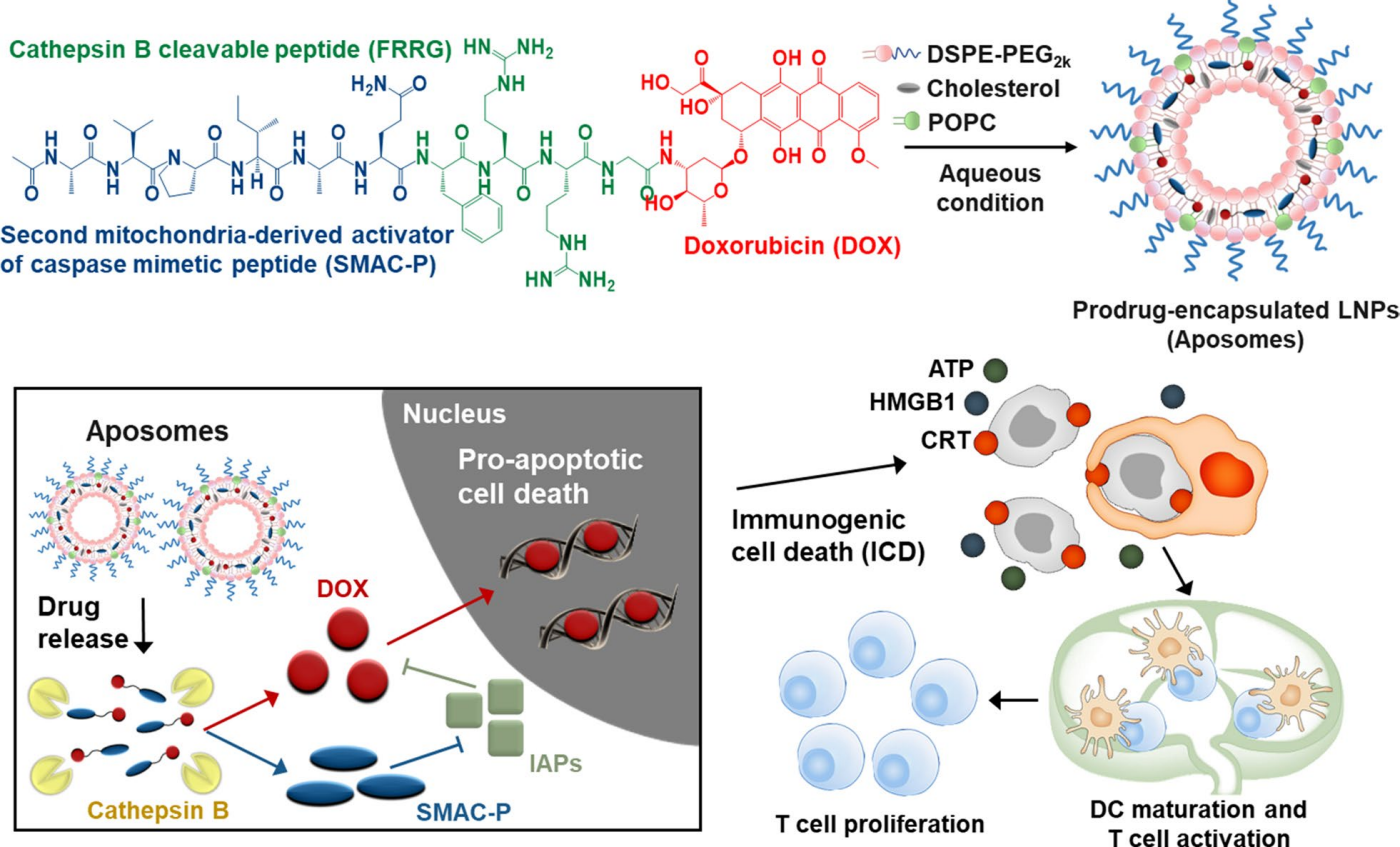

**Supplementary Information:**

The online version contains supplementary material available at 10.1186/s12951-024-02314-w.

## Background

Immunogenic cell death (ICD), one type of cell death in response to certain chemotherapeutic drugs in the cancer cells, is crucial approach in enhancing cancer immunogenicity of immunosuppressive tumor microenvironment (ITM) to turn into immune-responsive milieu [1]. Such cell death involves changes in the composition of the calreticulin (CRT) expression and the extracellular release of soluble mediators, such as high-mobility group box 1 (HMGB1), adenosine triphosphate (ATP) and heat shock protein 70 (HSP70) [2]. These signals from cancer cells undergoing ICD are called damage-associated molecular patterns (DAMPs), which promote the interaction with the host’s immune system to send ‘find me’ and ‘eat me’ signals and thereby result in dendritic cell (DC) maturation and T cell activation [3]. These cascade events increase the number of tumor-infiltrating lymphocytes (TILs) in the ITM, resulting in an immune-responsive milieu that is favorable to predict outstanding response to ICB therapies [4]. However, cancer cells are generally resistant to the intrinsic mitochondrial cell death pathway damaging DNA lesions due to the p53-mediated adaptations by inhibitors of apoptosis proteins (IAPs) upregulation during chemotherapy, diminishing antitumor immune responses by ICD-inducing chemotherapeutic drugs [5]. In contrast, the extrinsic cell death pathway that responds to death ligands from the immune system is typically intact in cancer cells, which can provide a promising avenue to exploit for the induction of effective ICD [5]. Importantly, the expression patterns and levels of DAMPs in the cancer cells can be variable according to the types of cell death pathways [6]. Therefore, a synergistic activity of intrinsic and extrinsic cell death pathways can be an alternative approach to induce a potent ICD in cancer cells and greatly improve the ICB therapy efficiency, ultimately leading to tumor eradication.

Second mitochondria-derived activator of caspase mimetic compounds (SMACs) that are ongoing extensive clinical studies for cancer therapy efficiently promote the apoptosis by directly interacting and degrading IAPs, which primarily control the extrinsic cell death pathway [7, 8]. Such compound can enhance the antitumor immune responses by ICD-inducing chemotherapeutic drugs since they significantly sensitize cancer cells to standard chemotherapy, resulting in enhanced ICD effects within cancer cells [9]. Importantly, it has been increasingly reported that SMACs have independent immunological effects in cancer treatment [10]. First, IAPs antagonism by SMACs produces a co-stimulatory signal to improve adaptive immune responses towards tumors by regulating the activation of alternative NF-κB pathway in immune cells [11]. In addition, SMACs stimulate DC maturation and T cell proliferation, activation and expansion [12]. Also, these SMACs-mediated immunological effects have shown to significantly increase the T cell activity in cancer vaccine approaches [13]. However, SMACs have intrinsic limitations, such as low cell permeability, poor in vivo stability and bioavailability [14]; thus, versatile and generalizable platform for delivery of them simultaneously with ICD-inducing chemotherapeutic drugs to targeted tumors is highly desired to potentiate ICB therapy via an effective and safe combination approach.

**Scheme 1.**
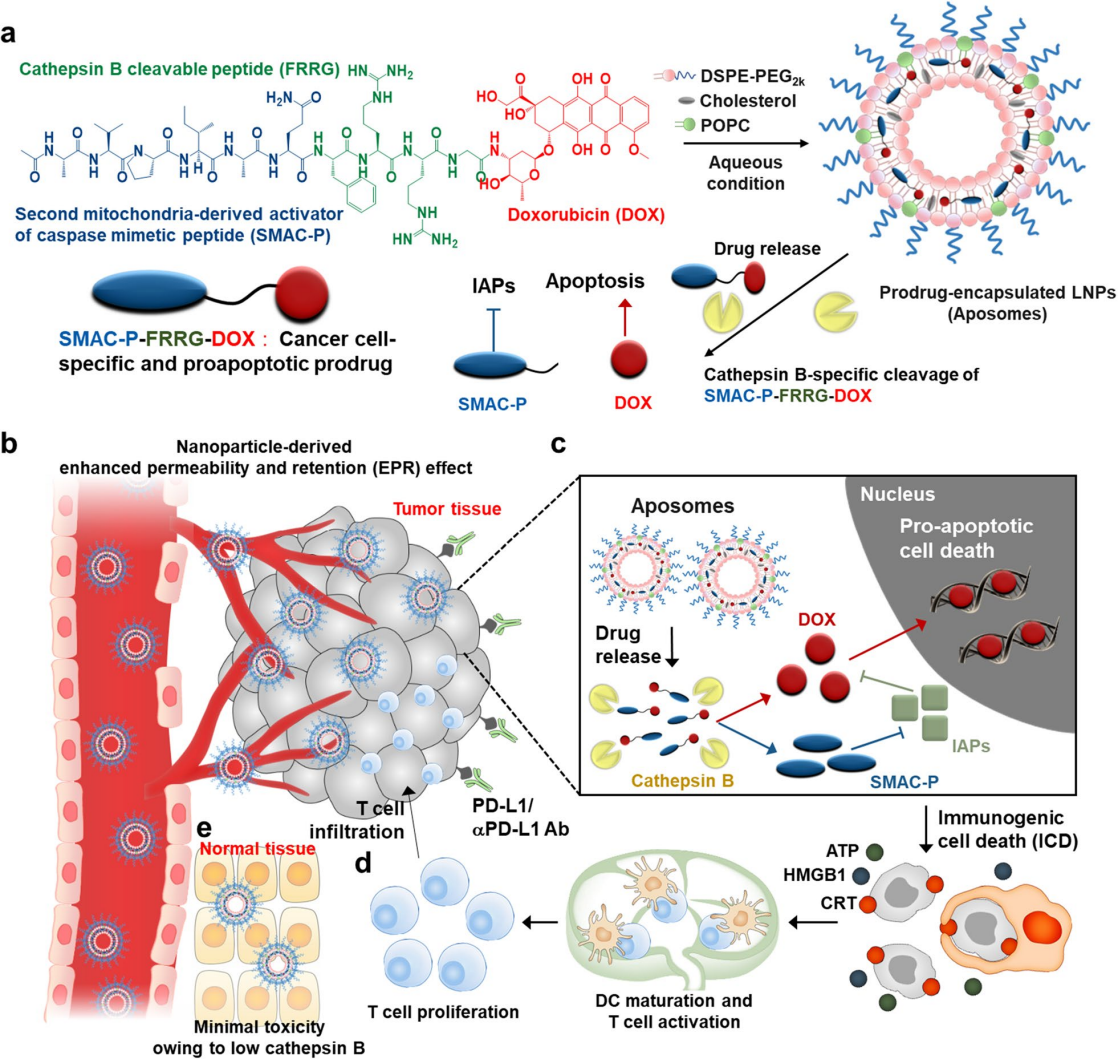
Synergistic activity of SMAC and DOX by Aposomes to potentiate ICB therapy. **a** Cancer cell-specific and pro-apoptotic prodrug of SMAC-P-FRRG-DOX, constructed with cathepsin B-cleavable SMAC-P (AVPIAQFRRG) and DOX, is formulated as clinically-relevant PEGylated liposomes, resulting in Aposomes. **b** In colon tumor bearing mice, Aposomes efficiently accumulate within the targeted tumor tissues via nanoparticle-derived EPR effect. **c** Prodrug of SMAC-P-FRRG-DOX is released from Aposomes and cleaved to SMAC-P and DOX by cathepsin B overexpressed in cancer cells. **d** The synergistic activity of SMAC-P and DOX induces a potent ICD accompanying DAMP expressions in cancer cells, resulting in DC maturation and T cell recruitment and activation and eventually eradicating the tumors. **e** Meanwhile, SMAC-P-FRRG-DOX released from Aposomes maintains the non-toxic inactive state in the normal tissues owing to lower cathepsin B, reducing the risk of complications. In same context, Aposomes also prevent the immunosuppression by systemic chemotherapy owing to minimized non-specific cytotoxicity to T cells, DCs and macrophages, which express a significantly low cathepsin B compared to cancer cells

Herein, we propose a cancer cell-specific and pro-apoptotic prodrug encapsulated PEGylated liposomes (Aposomes) inducing an effective ICD in the cancer cells to potentiate combinational ICB therapy. Previously, we developed a cancer cell-specific and pro-apoptotic prodrug of SMAC-P-FRRG-DOX that is consisted of second mitochondria-derived activator of caspases mimetic peptide (SMAC-P: *AVPIAQ*), cathepsin B-cleavable peptide (*FRRG*) and doxorubicin (DOX), which is specifically cleaved to SMAC-P and DOX in cathepsin B-overexpressed cancer cells. In drug-resistant breast tumor models, the SMAC-P-FRRG-DOX showed a significant antitumor efficacy owing to a synergistic activity of IAPs antagonism with chemotherapy. In present study, the SMAC-P-FRRG-DOX is further formulated as clinically-relevant PEGylated liposomes, and their potential ability in enhancing cancer immunogenicity of the ITM via synergistic activity of IAPs antagonism and ICD is evaluated (Scheme 1a). The additional formulation of prodrug nanoparticles as PEGylated liposomes enhances particle stability to improve the pharmacokinetic (PK) properties for more effective and safer ICB therapy. First, the intravenously injected Aposomes efficiently accumulate within the targeted tumor tissues via nanoparticle-derived EPR effect (Scheme 1b). Then, Aposomes are taken up by targeted cancer cells and the prodrug of SMAC-P-FRRG-DOX is released and cleaved to SMAC-P and free DOX in the cathepsin B-overexpressed cancer cells (Scheme 1c). Importantly, the synergistic activity of SMAC-P and free DOX induces a potent ICD accompanying DAMP expressions in cancer cells to promote DC maturation and T cell recruitment and activation, turning ITM into immune-responsive milieu and eventually eliciting antitumor immune responses (Scheme 1d). Therefore, the combination of Aposomes with anti-PD-L1 antibody leads to a complete tumor regression of the primary tumors and also prevent the recurrence by rechallenged tumors owing to immunological memory established during Aposomes treatment. Meanwhile, Aposomes maintain the inactive state in lower cathepsin B-expressed normal tissues, reducing toxic side effects and the risk of complications (Scheme 1e). In same context, Aposomes also prevent the immunosuppression by systemic chemotherapy via minimized non-specific cytotoxicity to T cells, DCs and macrophages, which express a significantly low cathepsin B compared to cancer cells. We demonstrate the synergistic activity of SMAC-P and DOX by Aposomes for ICD effect within the cancer cells, which are beneficial to improve the ICB therapy efficiency. The in vivo antitumor efficacy and immune responses of Aposomes in combination with anti-PD-L1 therapy are also assessed to confirm the adaptive immunity establishing systemic immunological memory in colon tumor models.

## Results and discussion

### Preparation of cancer cell-specific and pro-apoptotic Aposomes.

**Fig. 1:**
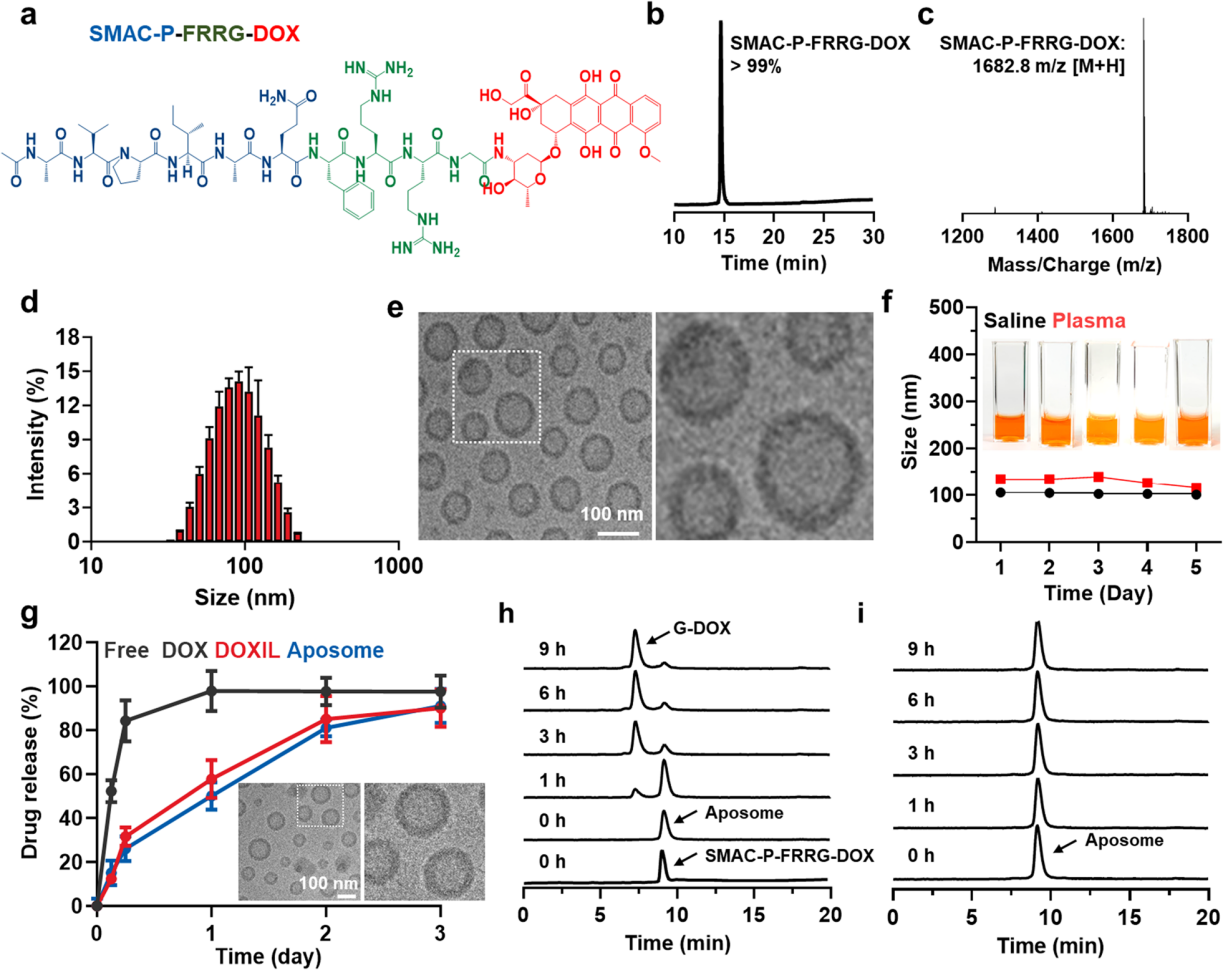
Preparation of cancer cell-specific and pro-apoptotic Aposomes. **a** Chemical structure of SMAC-P-FRRG-DOX. **b** Purity of prepared SMAC-P-FRRG-DOX. **c** MALDI-TOF analysis of SMAC-P-FRRG-DOX. **d** Size distribution of Aposomes in saline (1 mg/ml). **e** TEM image of Aposomes in distilled water (1 mg/ml). **f** Stability of Aposomes in saline or mouse plasma (1 mg/ml) for 5 days. The inset pictures indicate representative images of Aposomes in mouse serum conditions. **g** Release profiles of free DOX, DOXIL and Aposomes. TEM images reveal the particle size and morphology of Aposomes after 72 h of incubation in distilled water. **h** HPLC spectrum of Aposomes incubated with MES buffer (pH 5.5) containing cathepsin B (10 µg). As a control, SMAC-P-FRRG-DOX in MES buffer in the absence of cathepsin B was analyzed using an HPLC system.** (i)** HPLC spectrum of Aposomes incubated with cathepsin B with inhibitor (Z-FA-FMK)

As a clinically-relevant PEGylated liposomes to induce a potent ICD in the cancer cells via synergistic activity of SMAC peptide (SMAC-P) and DOX, Aposomes were prepared with two-step protocols of prodrug synthesis and liposome formation. First, the cathepsin B-cleavable SMAC-P, SMAC-P-FRRG (*Ala-Val-Pro-Ile-Ala-Gln-Phe-Arg-Arg-Gly*; AVPIAQFRRG), was conjugated to a DOX through one-step EDC/NHS reaction, resulting in SMAC-P-FRRG-DOX (Fig. 1a and Additional file 1: Figure S1). The AVPIAQ sequence was selected as a SMAC-P owing to its potent activity for IAP antagonism compared to other sequences, such as AVPIAQK, AVPIA and AVPI [15]. In addition, FRRG peptide sequence in the prodrugs is cleaved by target enzyme of cathepsin B overexpressed in cancer cells to trigger drug release with high cancer cell specificity in vitro and in vivo [16–19]. After the reaction, 99% of SMAC-P-FRRG-DOX was purified with reversed-phase high-performance liquid chromatography **(**RP-HPLC; Fig. 1b**)**. Furthermore, successful prodrug synthesis was confirmed via matrix-assisted laser desorption/ionization time-of-flight (MALDI-TOF) mass spectrometer, wherein the exact molecular weight was calculated to be 1681.87 Da for C_79_H_112_N_18_O_23_ and measured to be 1682.8 m/z [M + H] (Fig. 1c). ^1^H-NMR spectra also demonstrated characteristic peaks for the peptide and DOX in the 1.1–1.8 ppm and 6.8–8.4 ppm, respectively (Additional file 1: Figure S2). In order to prepare Aposomes, POPC, cholesterol, DSPE-PEG_2k_ and SMAC-P-FRRG-DOX were dissolved in chloroform at a specific molar ratio of 59.1:22.7:9.1:9.1 mol%, and the solution was evaporated to cast a uniform lipid film, followed by hydration with PBS for 30 min at 40  C. Under optimal condition, approximately 10 wt% of SMAC-P-FRRG-DOX was encapsulated into Aposomes with a 95% of drug loading efficiency. The prepared Aposomes showed a homogeneous lipid bilayer structure with an average size of 109.1 ± 5.14 nm, measured using the Intensity Distribution Mode (n = 3) **(**Fig. 1d**)**. The average size of Aposomes was little larger than the average size of 100 nm in the histogram. This is because the intensity distribution mode in DLS measurement can reflect the particle size larger than the number distribution in DLS measurement. TEM images showed a spherical morphology and a uniform size distribution of Aposomes **(**Fig. 1e**)**. Compared to conventional drugs loaded into the core of liposomes, amphiphilic prodrugs with a molecular weight exceeding 1000 kDa are considered to be complexed with the lipid bilayer [20, 21]. The zeta potential value of Apopsomes was measured to be -21.5 ± 2.21 mV, indicating a change to a negative charge compared to SMAC-P-FRRG-DOX with zeta potential of 14.9 ± 0.814 mV owing to the positively charged “RR” sequence (Additional file 1: Figure S3). In addition, Aposomes were highly stable in both saline and mouse plasma with no significant changes in particle size for 5 days **(**Fig. 1f**)**. The enhanced particle stability following formulation as PEGylated liposomes was clearly demonstrated when compared to SMAC-P-FRRG-DOX nanoparticles, which exhibited remarkable aggregation after 2 days of incubation in mouse plasma (Additional file 1: Figure S4). In addition, these stable nano-sized structures with approximately 100 nm is highly suitable to efficiently accumulate within the targeted tumor tissues via nanoparticle-derived EPR effect [22, 23]. Next, the drug release profile of Aposomes was compared to free DOX and DOXIL that is clinically-approved PEGylated liposomal formulation of DOX. In contrast to free DOX showing very fast release from dialysis membranes within 6 h, Aposomes showed a similar SMAC-P-FRRG-DOX release profiles in comparison to free DOX from DOXIL, with approximately 85% of each drug was slowly released for 3 days **(**Fig. 1g**)**. We have also demonstrated that Aposomes maintained their intrinsic particle size and morphology after 72 h of incubation in D.W, as confirmed by TEM images. These results indicate that the release mechanism of SMAC-P-FRRG-DOX is considered to occur through passive diffusion, in contrast to DOXIL, which releases free DOX due to a collapse of the liposome structure. Finally, the target enzyme-specific cleavage of SMAC-P-FRRG-DOX encapsulated in the Aposomes was assessed in vitro. Firstly, characteristic peaks of both Aposomes and SMAC-P-FRRG-DOX were similarly observed in the HPLC spectrum, indicating that the mixed solvent of H_2_O and ACN in HPLC system disassembled the liposome structure. When Aposomes (1 mg/ml) were incubated with cathepsin B (10 µg) in MES buffer (pH 5.5), a new HPLC peak of glycine-conjugated DOX (G-DOX) was clearly observed at the 7 min in the spectrum after 1 h of incubation, which was gradually increased up to 99% for 9 h post-incubation **(**Fig. 1h**)**. The LC/MS analysis confirmed the exact molecular weight of G-DOX (calculated: 600.98 Da, found: 623.3 m/z [M + Na]), but SMAC-P was not observed in HPLC spectrum due to its high hydrophilicity (Additional file 1: Figure S5). It was previously confirmed that SMAC-P-FRRG-DOX was cleaved to G-DOX and AVPIAQFR peptide that has comparable IAPs antagonism activity to AVPIAQ sequence after incubation with cathepsin B [24]. Furthermore, G-DOX cleaved from SMAC-P-FRRG-DOX can be metabolized into free DOX by intracellular proteases [25]. In contrast, cleavage behaviors of Aposomes were not observed for 9 h of incubation in the cathepsin B-suppressed condition in which target enzyme is pre-incubated with irreversible cathepsin B inhibitor, Z-FA-FMK **(**Fig. 1i**)**. We already confirmed that SMAC-P-FRRG-DOX is not cleaved by other enzymes such as, cathepsin E, D, L, caspase-3 and MMP-9 [24]. Taken together, Aposomes efficiently encapsulated SMAC-P-FRRG-DOX along with high stability in physiological condition as a clinically-relevant PEGylated liposome formulation. In addition, Aposomes could release the SMAC-P-FRRG-DOX to be cleaved to SMAC-P and DOX by target enzyme of cathepsin B in vitro*.*

### Cancer cell-specific and pro-apoptotic property of Aposomes in cell culture system

**Fig. 2:**
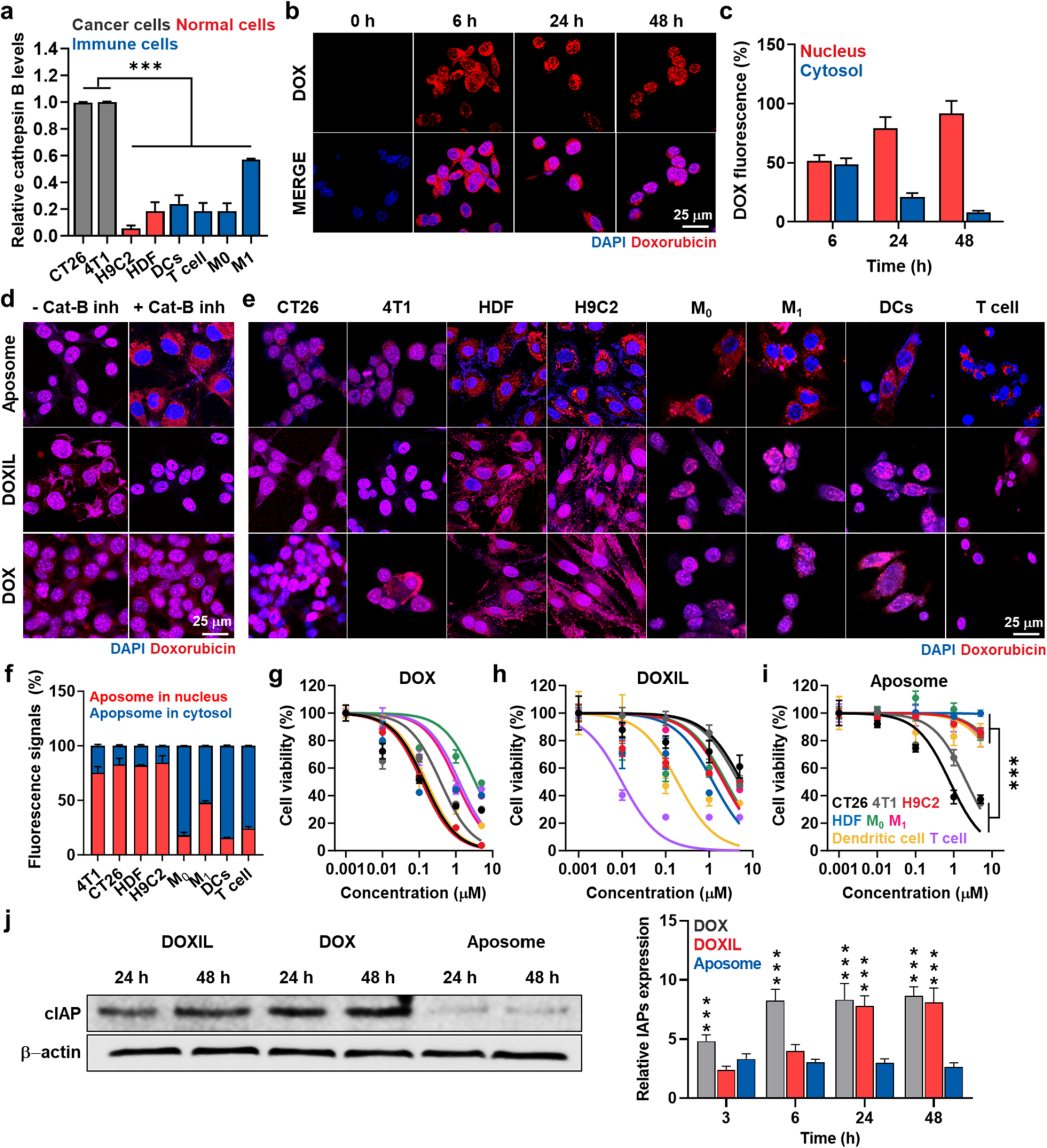
Cancer cell-specific cytotoxicity of Aposomes in cell culture system. **a** Relative expression of cathepsin B in CT26 and 4T1 cancer cells, H9C2 and HDF normal cells, and immune cells of M0 and M1 macrophages, DCs and T cells. **b** Time-dependent cellular uptake of Aposomes (2 µM based on DOX contents) in CT26 cells. **c** Relative DOX fluorescence in cytosol or nucleus of CT26 cells after treatment with Aposomes. **d** Fluorescence images of CT26 cells treated with free DOX, DOXIL or Aposomes (2 µM based on DOX contents) for 48 h with or without cathepsin B inhibitor, Z-FA-FMK. **e** Fluorescence images of CT26 and 4T1 cancer cells, H9C2 and HDF normal cells, and immune cells of M0 and M1 macrophages, DCs and T cells, which are treated with free DOX, DOXIL or Aposomes for 48 h. **f** Relative DOX fluorescence in cytosol or nucleus of CT26 and 4T1 cancer cells, H9C2 and HDF normal cells, and immune cells of M0 and M1 macrophages, DCs and T cells after treatment with Aposomes for 48 h. **g–i** Cell viability of CT26 and 4T1 cancer cells, H9C2 and HDF normal cells, and immune cells of M0 and M1 macrophages, DCs and T cells, which are treated with **g** free DOX, **h** DOXIL or **i** Aposomes for 48 h. **j** Expression levels of IAP in CT26 cells after treatment with free DOX, DOXIL or Aposomes for 48 h. Significance was determined by Tukey − Kramer post-hoc test

The cancer cell-specific and pro-apoptotic property of Aposomes premised on differential cathepsin B levels was assessed in cancer cells compared to other normal or immune cells. As expected, CT26 and 4T1 cancer cells expressed 3.41 ± 0.38-folds, 3.49 ± 0.42-folds, 6.01 ± 0.51-folds, 2.01 ± 2.22-folds, 3.22 ± 0.19-folds and 4.97 ± 0.34-folds of cathepsin B compared to normal cells of rat cardiomyocytes (H9C2) and human dermal fibroblast (HDF) as well as immune cells of M0 and M1 macrophages, DCs and T cells, respectively (Fig. 2a and Additional file 1: Figure S6). First, the intrinsic cellular uptake of Aposomes (2 µM based on DOX contents) according to incubation time was evaluated in the cathepsin B-overexpressed cancer cells. The time-dependent cellular uptake of Aposomes was clearly observed in cathepsin B-overexpressed CT26 cells, wherein a strong free DOX fluorescence signals (red color) of Aposomes (red color) increased rapidly for 6 h and then increased gradually over the next 48 h **(**Fig. 2b**)**. This indicated that Aposomes are taken up by cancer cells over time through a nanoparticle-derived cellular uptake mechanism [16]. Next, we carefully characterized the localization of free DOX within nucleus of cancer cells at independent times. It has been known that the prodrug of SMAC-P-FRRG-DOX in Aposomes cannot enter the nuclear membrane of cancer cell [24]. Importantly, SMAC-P-FRRG-DOX can enter the nucleus membrane only when it reacts with cathepsin B and is broken down into free DOX only in cathepsin B-overexpressed cancer cells. As we expect, after 6 h post-incubation, an almost 50:50 ratio of DOX fluorescence signals was observed in nucleus and cytoplasm of cancer cells **(**Fig. 2c**)**. However, when the incubation time increased to 48 h, the ratio of DOX florescence signals in the nucleus increased by 91.87% and the amount of DOX fluorescence signals in the cytoplasm decreased by 8.13%. From these cellular uptake studies, free DOX was successfully cleaved from SMAC-P-DOX and it was clearly observed in nucleus of cathepsin B-overexpressed cancer cells. Accumulation of free DOX molecules in the nuclei is essential to inhibit topoisomerase II activity with DNA, stopping the process of DNA replication [26]. Therefore, these results indicate that Aposomes efficiently internalize in the cancer cells to release SMAC-P-FRRG-DOX, which is eventually cleaved to SMAC-P and free DOX via cathepsin B-specific cleavage mechanism in cell culture system. The target enzyme-specific drug release of Aposomes was evaluated in the cathepsin B-suppressed CT26 cells that were pre-treated with irreversible cathepsin B inhibitor Z-FA-FMK for 24 h. In contrast to naive CT26 cells, free DOX signals in the nuclei were significantly decreased to the levels of 10% in the Z-FA-FMK-treated CT26 cells, indicating that un-cleaved SMAC-P-FRRG-DOX mainly localized in cytoplasm (Fig. 2d and Additional file 1: Figure S7). As a control, when both naive or Z-FA-FMK-treated CT26 cells were treated with free DOX or DOXIL, more than 90% of free DOX fluorescence was detected in the nuclei due to the rapid localization of free DOX molecules in the nuclei. Furthermore, given exceedingly low cathepsin B levels of H9C2, HDF, M0 and M1 macrophages, DCs and T cells, DOX fluorescence of Aposomes remained limited in the cytoplasm, wherein only less than 5% of those signals were observed in the nuclei after 48 h incubation (Fig. 2e, f). As a control, due to the relatively higher expression of cathepsin B in M1 macrophages than other normal and immune cells, half of fluorescence signals were detected within the nuclei. As expected, all the cell types treated with free DOX or DOXIL showed a strong DOX fluorescence signals in the nuclei regardless of their cathepsin B expression levels (Additional file 1: Figure S8). Additionally, the cellular uptake was quantitatively analyzed using flow cytometry, wherein DOXIL and Aposomes demonstrated a robust cellular uptake in all types of cancer, normal and immune cells through nanoparticle-derived endocytosis, with levels comparable to free DOX that exhibits fast cellular uptake due to a passive diffusion mechanism (Additional file 1: Figure S9). These different intracellular behaviors of Aposomes in the cancer cells compared to normal and immune cells led to the cancer-specific cytotoxicity.

Free DOX exhibited a strongest toxicity in cancer, normal and immune cells, with 0.003 to 0.38 μM of IC_50_ values in all the cell types owing to its rapid internalization and nucleus localization mechanism (Fig. 2g and Additional file 1: Figure S10). In contrast, DOXIL showed a relatively reduced cytotoxicity compared to free DOX because of the delayed drug release mechanism, with 0.19 to 5.66 μM of IC_50_ values in cancer, normal and immune cells (Fig. 2h and Additional file 1: Figure S10). Importantly, both free DOX and DOXIL did not show any cancer cell-specific cytotoxicity. Interestingly, Aposomes exhibited a significantly reduced cytotoxicity towards H9C2 (~ 15-folds), HDF (~ 400-folds), M0 macrophages (~ 15-folds), M1 macrophages (~ 10-folds), DCs (~ 10-folds) and T cells (~ 10-folds) compared to IC_50_ values in CT26 (0.8 µM) and 4T1 (2.16 µM) cancer cells (Fig. 2i and Additional file 1: Figure S10). Although M1 macrophages exhibit relatively higher levels of cathepsin B, a significant reduction in cytotoxicity of Aposomes was clearly observed compared to free DOX and DOXIL due to their cathepsin B levels still being relatively lower than cancer cells. Therefore, Aposomes can greatly reduce the clinical disadvantages of unwanted side effects by systemic chemotherapy, such as severe toxicity and inflammatory responses in normal organs, owing to a minimized lethal effect on normal cells. Furthermore, their reduced cytotoxicity against macrophages, DCs and T cells can prevent a systemic immunosuppression by immune cell loss and its dysfunction. Most importantly, the IC_50_ values of Aposomes were significantly lower to the levels of 14% and 45% than DOXIL in CT26 and 4T1 cells, respectively. This is attributable to the synergistic activity of SAMC-P and DOX owing to IAP antagonism to sensitize the cancer cells towards chemotherapy, which was clearly confirmed via additional western blot analyses. In case of CT26 cells treated with free DOX or DOXIL, intracellular IAPs expression levels was significantly upregulated (Fig. 2j and Additional file 1: Figure S11). These results indicate that cancer cells establish a resistance to the free DOX or DOXIL via upregulation of IAPs during chemotherapy, reducing the effects of intrinsic cell death pathway damaging DNA lesions. In contrast, Aposomes significantly inhibited the IAPs overexpression in the cancer cells compared to free DOX (0.34-folds) and DOXIL (0.32-folds) after 48 h of treatment, respectively.

### Immunogenic cell death (ICD) of Aposomes in cell culture system

**Fig. 3:**
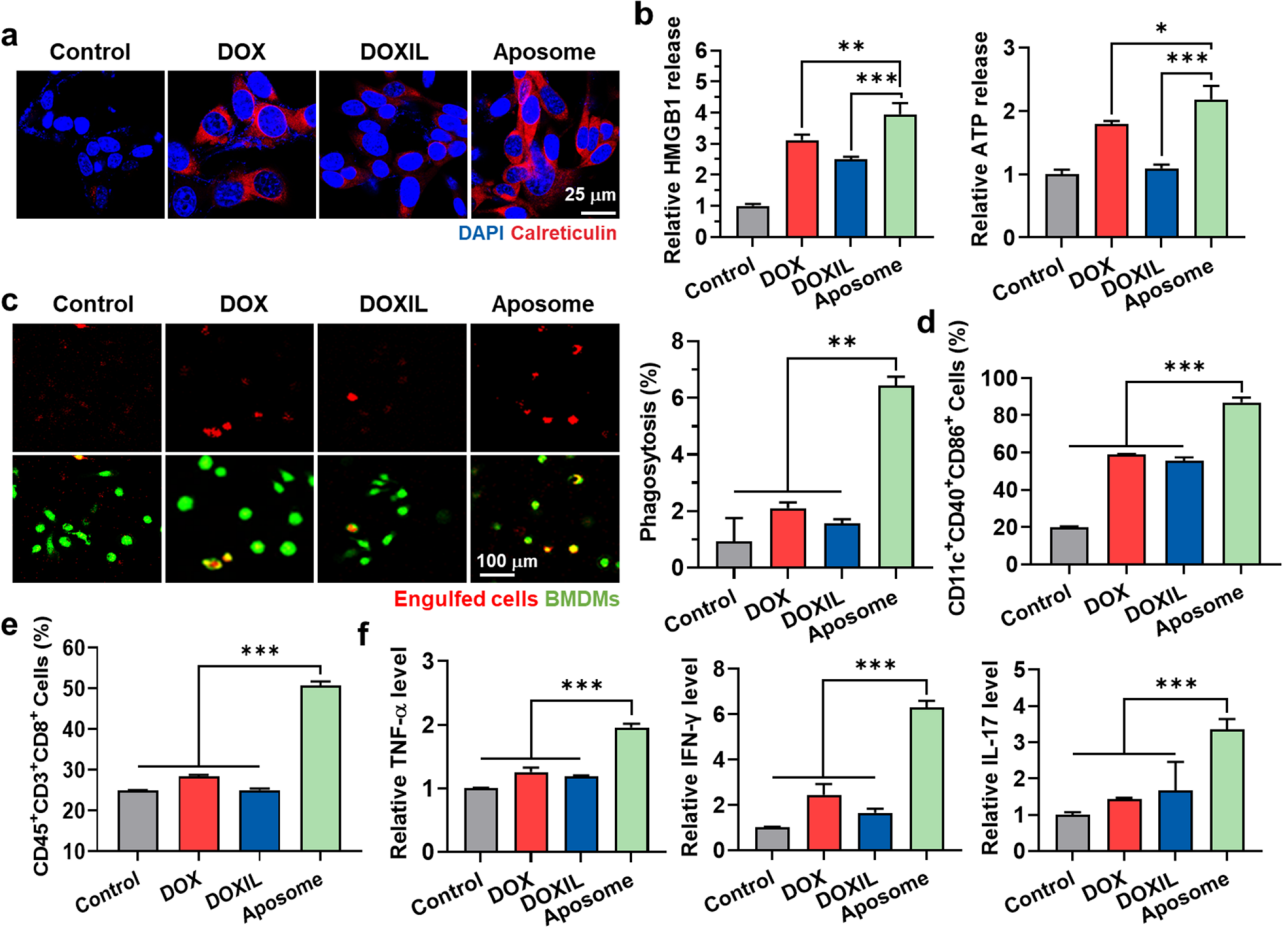
Immunogenic cell death (ICD) of Aposomes in cell culture system. **a** Fluorescence images CRT-stained CT26 cells after treated with free DOX, DOXIL or Aposomes (2 µM based on DOX contents) for 48 h. **b** Relative amount of HMGB1 and ATP released from CT26 cells treated with free DOX, DOXIL or Aposomes (2 µM based on DOX contents) for 48 h. **c** Fluorescence images of CellTracker Green-labeled BMDMs co-cultured with free DOX-, DOXIL- or Aposome-treated CT26 cells (pHrodo labeled) for 2 h. **d** Percentage of mature DCs (CD11c^+^CD40^+^CD86^+^) and **e** cytotoxic T cells (CD45^+^CD3^+^CD8^+^) within the lymphocytes after co-culture with CT26 cells treated with free free DOX, DOXIL or Aposomes for 48 h. **f** Relative amount of TNF-α, IFN-γ and IL-17 in the cell culture medium after co-culture of lymphocytes with CT26 cells treated with free free DOX, DOXIL or Aposomes for 48 h. Significance was determined by Tukey − Kramer post-hoc test

A potent ICD premised on synergistic activity of SMAC-P and DOX from Aposomes was investigated in cancer cells by measuring the amount of DAMPs, such as surface-exposed CRT (ecto-CRT) and extracellular release of HMGB1 and ATP. First, the ecto-CRT was assessed via confocal fluorescence imaging of CT26 cells treated with an equivalent DOX dose (2 µM) of free DOX, DOXIL or Aposomes for 48 h. The CRT fluorescence signals (red color) on the cell surface was significantly stronger in CT26 cells after treatment with Aposomes than free DOX or DOXIL **(**Fig. 3a**)**. As a control, CT26 cells treated with DOXIL revealed a substantially lower levels of ecto-CRT compared to those treated with free DOX, which is attributable to the delayed cellular uptake and drug release of liposomal formulation compared to rapid diffusion of free DOX. Therefore, these results also indicate that an ounstanding synergistic activity of SMAC-P and DOX released from Aposomes lead to a potent ICD in cancer cells in spite of their delayed cellular uptake and drug release in cell culture system. After treatment of Aposomes in CT26 cells, extracellular release of HMGB1 and ATP was also larger 2.01–2.08-folds and 2.05–2.24-folds, and 1.24–1.36-folds and 2.1–2.22-folds than those from cells treated with free DOX and DOXIL, respectively (Fig. 3b and Additional file 1: Figure S12). The effective ICD effects by Aposomes were further evaluated in co-culture assays. First, we confirmed a phagocytosis enhancement of macrophages by a high DAMPs from cancer cells treated with Aposomes. For these analyses, CT26 cells were treated with free DOX, DOXIL or Aposomes (2 µM based on DOX contents) for 48 h and subsequently labeled with a pH-sensitive dye, pHrodosuccinimidyl ester that emits the red fluorescence signals in the acidic phagosomes. Then, they were further co-cultured for 2 h with bone marrow-derived macrophages (BMDMs) labeled with CellTracker^™^ Green. The engulfment of cancer cells (red color) by macrophages (green color) was clearly observed through confocal fluorescence imaging, wherein the phagocytosis of CT26 cells by BMDMs was significantly higher after treatment with Aposomes (6.43 ± 0.31%) than free DOX **(**2.1 ± 0.21%) or DOXIL (1.57 ± 0.14%; Fig. 3c**)**. In addition, when the CT26 cells treated with Aposomes, free DOX or DOXIL were co-cultured with immature bone marrow-derived dendritic cells (BMDCs) for 2 h, the proportion of mature DCs (CD11c^+^CD40^+^CD86^+^) was greatly upregulated in the Aposome group compared to free DOX (1.52–1.63-folds) and DOXIL (1.61–1.69-folds) groups **(**Fig. 3d**)**. A significant effect for T cell proliferation and activation was also confirmed by observing a high population of CD45^+^CD3^+^CD8^+^ cells in the lymphocytes and increase in secreation of TNF-α (1.68–1.74-folds and 1.71–1.77-folds), IFN-γ (2.49–2.64-folds and 3.68–3.81-folds) and IL-17 (2.22–2.57-folds and 1.42–3.55-folds) after co-culture with CT26 cells treated with Aposomes than free DOX or DOXIL (Fig. 3e, f). A significant increase of certain cytokines, such as TNF-α, IFN-γ and IL-17, indicate a robust CD8^+^ cell response premised on synergistic activity of SMAC-P with free DOX [27]. These results demonstrate that synergistic activity of SMAC-P and DOX by Aposomes can lead to the effective ICD in the cancer cells, modulating the multiple steps related to the cancer-immunity cycle and ultimately promoting a strong antitumor immune response.

### Tumor targeting of Aposomes in colon tumor models

**Fig. 4:**
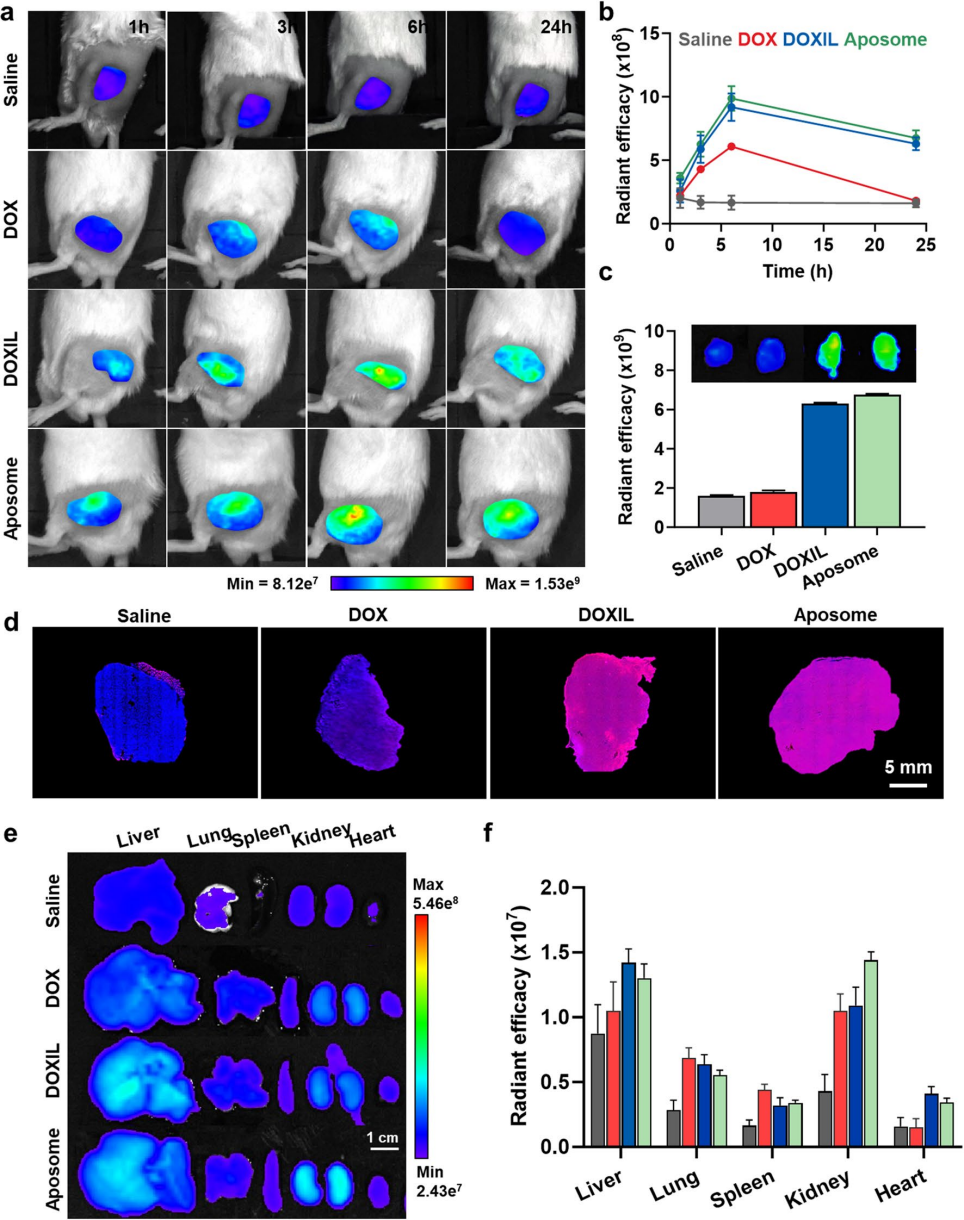
Tumor targeting of Aposomes in colon tumor models. **a** NIRF images of CT26 colon tumor-bearing mice after treatment with saline or an equivalent DOX dose (3 mg/kg) of free DOX, DOXIL or Aposomes. **b** Quantitative analyses for DOX fluorescence signals within the tumor regions of CT26 colon tumor-bearing mice after treatment with saline or an equivalent DOX dose (3 mg/kg) of free DOX, DOXIL or Aposomes. **c** Fluorescence images and quantitative analyses of exercised tumor tisseus from CT26 colon tumor-bearing mice after treatment with saline or an equivalent DOX dose (3 mg/kg) of free DOX, DOXIL or Aposomes for 24 h. **d** Histological analyses of tumor tissues from CT26 colon tumor-bearing mice after treatment with saline or an equivalent DOX dose (3 mg/kg) of free DOX, DOXIL or Aposomes for 24 h. **e**, **f** Ex vivo fluorescence images of CT26 colon tumor-bearing mice after treatment with saline or an equivalent DOX dose (3 mg/kg) of free DOX, DOXIL or Aposomes. Significance was determined by Tukey − Kramer post-hoc test

To evaluate the enhanced pharmacokinetic (PK) properties of SMAC-P-FRRG-DOX after formulation as a PEGylated liposome, equivalent doses of 3 mg/kg based on DOX content for free DOX, SMAC-P-FRRG-DOX and Aposomes were intravenously injected into BALB/c mice, followed by the collection of blood samples at pre-determined time points (Additional file 1: Figure S13). Importantly, free DOX exhibited fast in vivo excretion with lower area under the curves (AUC) and C_max_, and high clearance (CL), whereas SMAC-P-FRRG-DOX demonstrated remarkably improved PK parameters. Notably, Aposomes showed significantly enhanced PK properties with 64.7-fold and 11.04-fold, 202.15-fold and 4.65-fold and 0.02-fold and 0.09-fold of AUC, C_max_ and CL compared to free DOX and SMAC-P-FRRG-DOX, respectively. The tumor targeting of Aposomes was next monitored in colon tumor-bearing mice, which are prepared by subcutaneous inoculation of 1 × 10^6^ CT26 cells into left flank of BALB/c mice. When the colon tumor volumes were approximately 200 ± 10 mm^3^, an equivalent DOX dose (3 mg/kg) of free DOX, DOXIL or Aposomes was intravenously injected into mice, and DOX fluorescence intensities were quantitatively analyzed using non-invasive near-infrared fluorescence (NIRF) imaging. As a control, only a small amount of free DOX was accumulated in tumor tissue and the accumulated amount did not increase after 6 h due to the low tumor targeting of small molecule drug (Fig. 4a). However, liposomal formulation of Aposomes and DOXIL showed a significantly increased DOX fluorescence signals in tumor tissues after 3 h of injection compared to auto-fluorescence signals from saline group, reaching their highest fluorescence intensities after 6 h post-injection via nanoparticle-derived EPR effect. Furthermore, both Aposomes and DOXIL accumulated in tumor tissues were sustainably retained for 24 h **(**Fig. 4b**)**. In addition, the ex vivo fluorescence imaging of excised tumor tissues further confirmed that tumor accumulation of Aposomes was significantly higher than that of free DOX and similar with DOXIL at 24 h post-injection **(**Fig. 4c**).** Quantitatively, the DOX fluorescence signals of Aposomes and DOXIL in the tumor tissues was approximately 2.06–2.32-folds stronger than those of free DOX. Immunohistochemistry (IHC) showed an even distribution of Aposomes and DOXIL (red color) in the whole area of tumor tissues after 24 h of injection, but less amount of free DOX was observed in the excised tumor tissues (Fig. 4d and Additional file 1: Figure S14). Furthermore, non-specific accumulation of Aposomes and DOXIL in liver tissues was clearly observed, wherein the liver accumulation of both liposomal formulations was 1.6–1.66-folds higher than free DOX (Fig. 4e, f). As a control, the saline group exhibited only weak auto-fluorescence signals in major organs. It is reported that liposomal formulation with an average size of 100 nm are readily removed by the reticuloendothelial system (RES) in liver [28]. Nevertheless, a significant toxicity of prodrugs towards normal organs was not observed owing to innately low cathepsin B expression in previous study, whereas DOXIL might cause severe side effects because of free DOX release [24, 29]. Finally, all DOX molecules in Aposomes might be excreted through the kidney in which the bright fluorescence signals was clearly observed at 24 h post-incubation. Additionally, significant tumor accumulation of Aposomes was further demonstrated in 4T1 breast tumor-bearing mice, indicating outstanding tumor-targeting ability across various tumor types (Additional file 1: Figure S15). Taken together, Aposomes efficiently accumulate within the targeted tumor tissues via a nanoparticle-derived EPR effect in colon tumor-bearing mice.

### Antitumor efficacy and immune response of Aposomes in colon tumor models

**Fig. 5:**
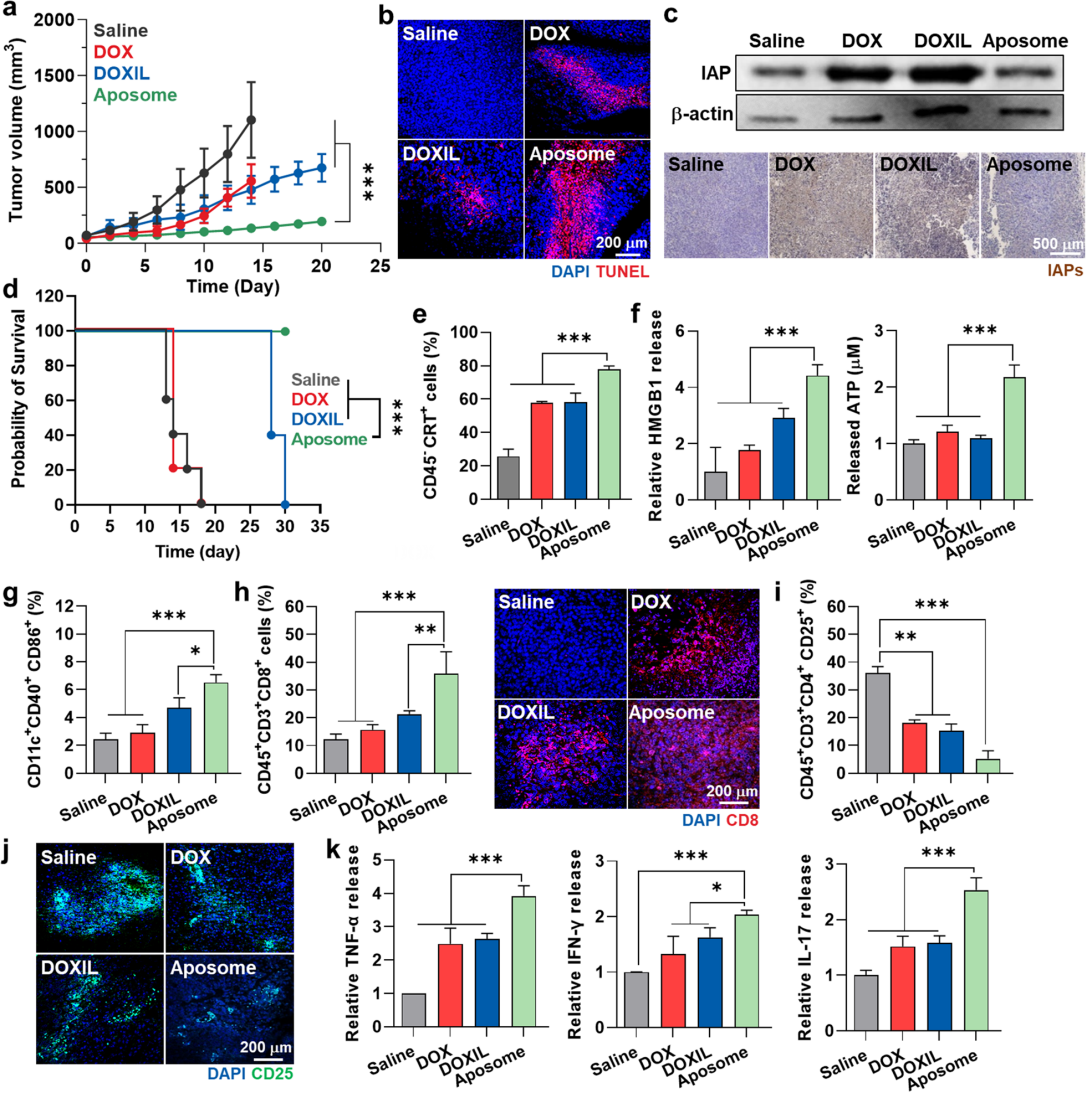
Antitumor efficacy and immune response of Aposomes in the colon tumor models. **a** Tumor growth curves of CT26 colon tumor-bearing mice during treatment with an equivalent 3 mg/kg DOX dose of free DOX, DOXIL or Aposomes once every three days. **b** Tumor tissues stained with TUNEL after 13 days of treatment. **c** Expression levels of IAP in cancer cells after 13 days of treatment with free DOX, DOXIL or Aposomes. **d** Survival of mice during treatment with an equivalent 3 mg/kg DOX dose of free DOX, DOXIL or Aposomes once every three days. **e** The percentage of PD-L1^+^ tumor cells (CD45^−^PD-L1^+^) in the tumor tissues on day 13 after treatment.** (f)** The amount of HMGB1 and ATP in tumor supernatants on day 13 after treatment. **g–j** The percentage of **g** mature DCs (CD11c^+^CD40^+^CD86^+^), **h** cytotoxic T cells (CD45^+^CD3^+^CD8^+^) and **i**, **j** regulatory T cells (CD45 + CD3 + CD4 + CD25 +) in the tumor tissues on day 13 after treatment.** k** Relative amount of TNF-α, IFN-γ and IL-17 in tumor supernatants on day 13 after treatment. Significance was determined by Tukey − Kramer post-hoc and log-rank tests

The in vivo antitumor efficacy and immune response of Aposomes were investigated in CT26 colon tumor models, which are widely utilized to evaluate antitumor immune responses due to its demonstration of changes in immune responses, including immunogenic cell death, tumor-infiltrating lymphocytes and cytokine release. The mice were randomly divided into four groups of (i) saline, (ii) free DOX, (iii) DOXIL and (iv) Aposomes, and an equivalent DOX dose (3 mg/kg) of each drug was intravenously injected once every three days, starting when the tumor volumes were approximately 50 ± 30 mm^3^. The antitumor efficacy was assessed by monitoring the growth of tumor volumes during treatments. Importantly, the Aposome group (n = 5, 133.86 ± 26.03 mm^3^) showed a significantly delayed tumor growth on day 14 compared to free DOX (n = 5, 556.2 ± 149.82 mm^3^), DOXIL (n = 5, 478.3 ± 126.52 mm^3^) and saline (n = 5, 1102.13 ± 337.98 mm^3^) groups (Fig. 5a and Additional file 1: Figure S16). In the case of free DOX, 4 out of 5 mice died within 13 days, due to its severe toxicity. TUNEL-stained tumor tissues exhibited an extensive red colored apoptosis after 13 days of Aposome treatment than the other treatments, demonstrating in vivo synergistic activity of SMAC-P and DOX **(**Fig. 5b**)**. Those synergistic activities were further confirmed in tumor tissues via immunohistochemistry (IHC) and western blot analysis on day 13 after treatment (Fig. 5c, Additional file 1: Figure S17). The treatment with free DOX or DOXIL led to an IAPs overexpression in the tumor tissues, resulting in resistance to the intrinsic mitochondrial cell death pathway by anthracyclines [30]. Unlike cell culture system, the negative feedback of IAPs expression according to DOXIL treatment in the tumor tissues was even greater than free DOX because of their high tumor accumulation. In contrast, Aposomes effectively inhibited the IAPs overexpression in tumor tissues during treatments by co-delivery of DOX with SMAC-P, wherein the levels of IAPs in the tumor tissues were significantly lower as levels of 53% and 45% than free DOX and DOXIL groups, respectively. Based on biocompatible liposomal formulation and high cancer selectivity of prodrugs loaded in the system, a notable body weight loss and structural abnormalities in major organs were not observed in the mice during Aposome treatments, whereas mice treated with free DOX exhibited a significant body weight loss along with extensive tissue damages due to its severe systemic toxicities (Additional file 1: Figure S18). As a result, the median survival of mice treated with saline, free DOX or DOXIL was determined to be 14, 14 and 28 days, respectively, while mice in the Aposome group survived over 30 days (Fig. 5d).

Next, the antitumor immune response was evaluated on day 13 after treatment with same protocol as described above. As expected, Aposome treatment significantly upregulated the percentage of CRT-positive tumor cells (CD45^−^CRT^+^; 77.87 ± 1.97%) within the tumor tissues compared to free DOX (57.67 ± 0.91%), DOXIL (58.3 ± 5.23%) or saline (25.7 ± 4.24%) groups **(**Fig. 5e**)**. Extracellular release of HMGB1 and ATP into tumor supernatants was also greatly increased in the Aposome group than other groups **(**Fig. 5f**)**. As a result of a potent ICD accompanying high DAMPs, the population of mature DCs (CD11c^+^CD40^+^CD86^+^) and cytotoxic T cells (CD45^+^CD3^+^CD8^+^) within the tumor tissues was 1.71–2.03-folds and 1.5–1.64-folds, and 2.41–2.66-folds and 2.32–2.44-folds higher in mice treated with Aposomes compared to those treated with free DOX and DOXIL, respectively (Fig. 5g, h). In addition, Aposome treatment significantly downregulated the population of Tregs (CD3^+^CD4^+^CD25^+^) within the tumor tissues, increasing the ratio of cytotoxic T cells to Tregs and eventually turning ITM into immune-responsive milieu that is favorable to predict remarkable efficacy with a high response rate to ICB therapy (Fig. 5i, j**)**. Finally, the high activity of cytotoxic T cells within the tumor tissues in the Aposome group was confirmed by measuring an increase of TNF-α, IFN-γ and IL-17 released from activated cytotoxic T cells to the tumor microenvironment **(**Fig. 5k**)**. Consequentially, synergistic activity of SMAC-P and DOX by Aposomes induces a potent ICD within the tumor tissues to recruit a large amount of cytotoxic T cells and exclude the Tregs in the ITM, resulting in immune-responsive milieu.

### Combination of Aposomes and ICB therapy in the colon tumor models

**Fig. 6:**
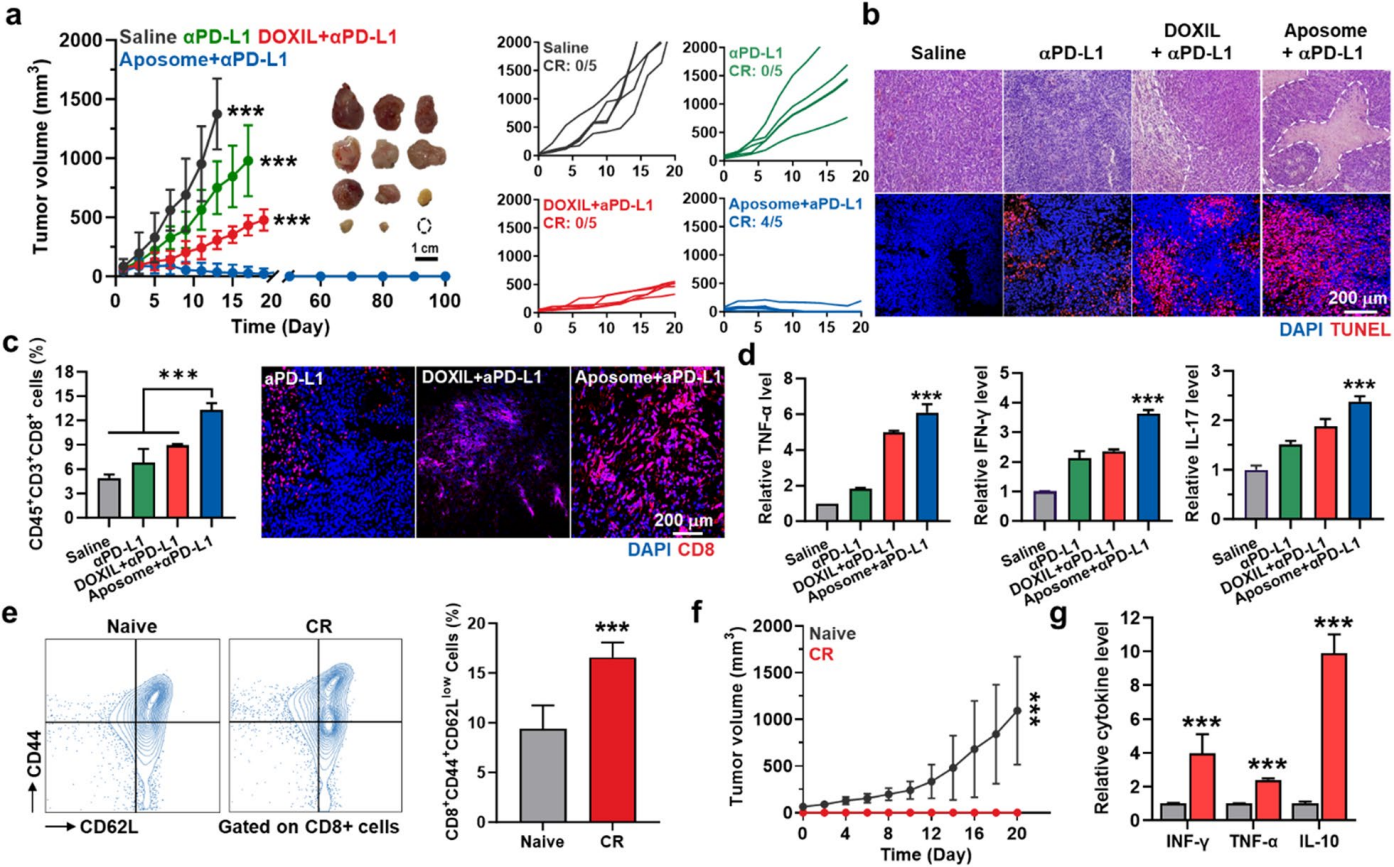
Combination of Aposomes and ICB therapy in the colon tumor models. **a** Tumor growth curves of CT26 colon tumor-bearing mice during treatment with DOXIL plus αPD-L1 Ab, Aposomes plus αPD-L1 Ab or αPD-L1 Ab only. Aposomes and DOXIL were injected with an equivalent 3 mg/kg DOX dose once every three days, and αPD-L1 Ab (10 mg/kg) was simultaneously administered via tail vein. **b** Tumor tissues stained with TUNEL of H&E after 13 days of treatment. **c** The percentage of cytotoxic T cells (CD45^+^CD3^+^CD8^+^) in the tumor tissues on day 13 after treatment. **d** Relative amount of TNF-α, IFN-γ and IL-17 in tumor supernatants on day 13 after treatment. **e** The percentage of splenic effector/memory CD8^+^ T cells (CD8^+^CD44^+^CD62L^low^) in CR and naive mice. **f** Tumor growth curves in naive and CR mice rechallenged with CT26 cells. **g** Cytokine levels in plasma on day 20 after CR mice were rechallenged with secondary tumors compared to naive mice. Significance was determined by Tukey − Kramer post-hoc test

To evaluate whether the synergistic activity of SMAC-P and DOX by Aposomes leads to potentiate the ICB therapy, CT26 tumor-bearing mice were randomly divided into three groups of (i) saline (n = 5), (ii) anti-PD-L1 antibody (αPD-L1 Ab; n = 5), (iii) DOXIL plus anti-PD-L1 antibody (n = 5) and (iv) Aposomes plus αPD-L1 Ab (n = 5). The mice were treated with an equivalent DOX dose (3 mg/kg) of DOXIL or Aposomes once every three days, and αPD-L1 Ab (10 mg/kg) was simultaneously injected through tail vein. As expected, Aposomes plus αPD-L1 Ab (35.91 ± 71.92 mm^3^) significantly inhibited the tumor growth compared to saline (1374.43 ± 300.64 mm^3^), αPD-L1 Ab (751.57 ± 224.07 mm^3^) and DOXIL plus αPD-L1 Ab groups **(**307.12 ± 77.31 mm^3^; Fig. 6a**)**. Tumor tissues stained with H and E or TUNEL also showed extensive structural abnormalities and apoptosis on day 13 after treatment with Aposomes plus αPD-L1 Ab compared to other treatments **(**Fig. 6b**)**. In particular, Aposomes plus αPD-L1 Ab group exhibited a high rate of complete tumor regression (CR: 4/5) up to 100 days, demonstrating a significantly potentiated ICB therapy. We have also observed the increase of T cells predominantly distributed in the tumor tissues from mice treated with Aposomes plus αPD-L1 Ab, wherein the levels of TNF-α, IFN-γ, IL-17 in the tumor supernatants were significantly upregulated owing to a T cell’s high activity (Fig. 6c, d**)**. This is attributable to significant immunological effects of SMAC-P that regulates the activation of alternative NF-κB pathway in immune cells as well as involves in stimulation of DC maturation and T cell proliferation [11, 12]. Next, the establishment of immunological memory that prevents the recurrence and metastasis from previously encountered cancer cells was investigated in the mice, which experienced CR by treatment with Aposomes plus αPD-L1 Ab. The effector/memory T cell (T_em_; CD3^+^CD8^+^CD44^+^CD62L^low^), a hallmark of adaptive immunity following cancer immunization, was significantly increased by a remarkable downregulation of CD62L in the splenic CD8^+^ T cells of CR mice (16.57 ± 1.5%) compared to naive mice **(**9.38 ± 2.37%; Fig. 6e**)**. In addition, CR mice were resistant to rechallenged tumor progression for 21 days when they were further experienced subcutaneous inoculation with CT26 cells on day 100 after Aposomes plus αPD-L1 Ab treatment **(**Fig. 6f**)**. These results clearly demonstrated long-term remission owing to the establishment of immunological memory against previously encountered tumor cells. Finally, the levels of cytokines including IFN-γ, TNF-α and IL-10 in the plasma were significantly upregulated in the CR mice compared to naive mice on day 21 after tumor rechallenge **(**Fig. 6g**)**. Finally, the antitumor efficacy of combination of Aposomes and αPD-L1 Ab was further assessed in 4T1 breast tumor-bearing mice, a relatively poor immunogenic tumor model [31]. Importantly, treatment with Aposomes and αPD-L1 Ab demonstrated a remarkable effect in inhibiting 4T1 tumor progression compared to free DOX, DOXIL and Aposomes (Additional file 1: Figure S19). Subsequent analyses also confirmed a significantly enhanced induction of ICD and upregulated cytotoxic T cells within the 4T1 tumor tissues, while simultaneously reducing T_reg_ cells, ultimately improving the survival of mice compared to other groups. These results clearly indicate that Aposomes greatly potentiate the ICB therapy by turning ITM into immune responsive milieu via synergistic activity of SMAC-P and DOX to induce a potent ICD and also prevent tumor recurrence by immunological memory established during combination treatment with αPD-L1 Ab.

## Conclusions

In this study, cancer cell-specific and pro-apoptotic prodrug encapsulated Aposomes were proposed to potentiate the ICB therapy in the ITM. Aposomes, designed as PEGylated liposomes formulating the cancer cell-specific and pro-apoptotic prodrug of SMAC-P-FRRG-DOX, efficiently promoted a SMAC-P-mediated IAPs antagonism in the manner of extrinsic cell death. We previously reported the development of SMAC-P-FRRG-DOX as an alternative nano-formulation and are currently undertaking preclinical studies for clinical translation. The rationale behind additional formulating them as PEGylated liposomes is to enhance the stability of nanoparticles, thereby improving PK properties for more effective and safer cancer immunotherapy. In fact, SMAC-P-FRRG-DOX nanoparticles exhibited significant aggregation from 48 h post-incubation in mouse serum. However, their particle stability markedly improved, with no significant changes in size for 5 days after formulation as PEGylated liposomes. As a result, Aposome treatment efficiently removed the resistance mechanism of cancer cells to intrinsic cell death pathway and thereby enhanced the ICD effects via synergy of SMAC-P and DOX. In colon tumor models, Aposomes passively targeted the tumor tissues via EPR effect and released the SMAC-P-FRRG-DOX, which was subsequently converted to SMAC-P and DOX by cathepsin B-overexpressed cancer cells. Then, the synergistic activity of SMAC-P and DOX induced a potent ICD in the cancer cells to promote a large number of TIL recruitment along with high DC maturation and T cell proliferation and activation; notably, the efficacy for antitumor immune responses was significantly higher compared to DOXIL, which is a representative drug delivery system currently used in clinical practice. These cascade events within the ITM by Aposomes prompted an immune-responsive tumor microenvironment that is favorable to predict outstanding response to ICB therapy. Finally, Aposome treatment in combination with anti-PD-L1 antibody efficiently eradicated the tumors with a high rate of complete regression and also effectively established in vivo immunological memory to prevent the tumor recurrence. This study highlighted a great potential to potentiate the ICB therapy by promoting a synergistic activity of SMAC-P and DOX via Aposomes. Furthermore, these formulations are highly suitable for encapsulating additional therapeutics for combinational cancer immunotherapy, which has the potential to address the formidable challenges faced in current cancer immunotherapy, particularly in delivering multiple drugs to targeted tumor tissues.

## Methods

### Reagents

N-terminal acylated SMAC-P-FRRG (AVPIAQFRRG) peptide was purchased from Peptron. (Daejeon, Republic of Korea). 1-palmitoyl-2-oleoyl-*glycero*-3- phosphocholine (POPC), 1,2-distearoyl-*sn*-glycero-3-phosphoethanolamine-*N*-[methoxy (polyethylene glycol)2 k] (DSPE-PEG_2k_) and DOXIL were purchased from Avanti Polar Lipids (Alabaster, AL, USA). N-hydroxysuccinimide (NHS), 1-ethyl-3-(3-dimethylaminopropyl)carbodiimide (EDC), hematoxylin and eosin (H&E) staining solution, acetonitrile (ACN) and dimethylformamide (DMF) were purchased from Sigma-Aldrich (St. Louis, MO, USA). Doxorubicin hydrochloride (DOX) was purchased form FutureChem (Seoul, Republic of Korea). TEM grid (Carbon Film 200 Mesh copper) was purchased from Electron Microscopy Sciences (PA, USA). Cathepsin B enzyme and IFN-γ Quantikine ELISA Kit (cat# SMIF00) were purchased from R&D systems (Minneapolis, MN, USA). Monoclonal anti-cathepsin B antibody and benzyloxycarbonyl-Phe-Ala-fluoromethylketone (Z-FA-FMK) were purchased from Santa Cruz Biotechnology (Dallas, Texas, USA). BCA protein quantification kit and streptavidin–horseradish peroxidase (streptavidin-HRP) were purchased from Thermo Fisher Scientific (Rockford, IL, USA). The antibodies against inhibitor of apoptosis proteins 1 and 2 (cIAP1/2), cleaved poly ADP ribose polymerase-1 (PARP-1), cleaved caspase-3 and beta-actin were purchased from Abcam (Hanam, Republic of Korea). RPMI 1640 media and antibiotics (streptomycin and 100 U/mL penicillin) were purchased from WELGENE Inc. (Daegu, Republic of Korea). Cell counting kit-8 (CCK-8) was purchased from Vitascientific (Beltsville, MD, USA). Fluorescent dye-conjugated antibodies against mouse CD45.2 (cat# 109828), mouse CD8a (cat# 100712), mouse CD3 (cat# 100218), mouse CD25 (cat# 126404) and mouse CD4 (cat# 100412) were purchased from BioLegend (San Diego, CA, USA). Tumor dissociation kit (cat# 130-096-730) was purchased from Miltenyi Biotechnoloy (Bergisch Gladbach, Germany). CT26 (mouse colon carcinoma cell line) was purchased from American Type Culture Collection (ATCC; Manassas, VA, USA).

### Preparation of cancer-specific and pro-apoptotic prodrug encapsulated Aposomes

First, cancer-specific and pro-apoptotic prodrug of SMAC-P-FRRG-DOX was synthesized through a one-step EDC/NHS reaction of AVPIAQFRRG peptide and DOX. The AVPIAQFRRG peptide (1.59 g, 1.375 mmol), EDC (1 g, 5.2 mmol), NHS (500 mg, 4.33 mmol) and DOX (1 g, 1.84 mmol) were dissolved in 200 mL of DMF. After 24 h of reaction at room temperature, the SMAC-P-FRRG-DOX was purified using Sep-Pak C18 column (Waters, Massachusetts, USA) and the resulting filtrates were analyzed using high performance liquid chromatography (HPLC, Agilent 1200 Series). The successful synthesis of SMAC-P-FRRG-DOX was confirmed by measuring exact molecular weight by using matrix-assisted laser desorption/ionization time of flight mass spectrometer (MALDI-TOF, AB Sciex TOF/TOF 5800 System, USA) with cyano-4-hydroycinnamic acid (CHCA) matrix. The chemical structure of SMAC-P-FRRG-DOX was characterized using 400 MHz ^1^H-NMR (DD2 FT NMR, Agilent Technologies, USA) after dissolving in DMSO-*d*_6_. Finally, the purified products were lyophilized to obtain as a red powder. Next, the Aposomes were prepared by thin-film hydration methods as described elsewhere. Briefly, POPC, cholesterol, DSPE-PEG_2k_ and SMAC-P-FRRG-DOX were dissolved in chloroform at a specific molar ratio of 59.1:22.7:9.1:9.1 mol%. Then, the solution was evaporated under a gentle stream of argon (Ar) at 60 °C to cast a uniform lipid film, which was subsequently hydrated with PBS at 40 °C for 20 min. Finally, the hydrated Aposomes were sonicated for 2 min under ice bath using a probe-type sonifier. The size distribution and zeta potential of Aposomes in saline (1 mg/ml) were confirmed by zeta-sizer (Nano ZS, Malvern Instruments, Worcestershire, UK), wherein the average size was measured using the intensity distribution mode. The size distribution and zeta potential of SMAC-P-FRRG-DOX and Aposomes in saline (1 mg/ml) were confirmed by zeta-sizer (Nano ZS, Malvern Instruments, Worcestershire, UK). The morphology of Aposomes was characterized in distilled water (1 mg/ ml) using transmission electron microscopy (TEM, CM-200, Philips, USA). The drug loading capacity of Aposomes was calculated based on DOX fluorescence of prodrugs after dissolving them in DMSO; consequently, the drug loading capacity was determined to be approximately 10%, similar to that of DOXIL, as provided by the manufacturer. Target enzyme-specific cleavage of Aposomes was monitored via HPLC. For this analysis, Aposomes (1 mg/ml) were incubated with MES buffer (pH 5.5) containing cathepsin B enzyme (10 µg) at 37 °C for 0, 1, 3, 6, 9 h, followed by analysis using HPLC with solvent gradient under H_2_O and ACN.

### Western blot

The levels of cathepsin B in different cell types were evaluated as described in our previous study [37]. Briefly, 5 × 10^5^ CT26 and 4T1 cancer cells, H9C2 and HDF normal cells, and immune cells of M0 and M1 macrophages, DCs and T cells were seeded into 6-well cell culture plates. Bone marrow-derived macrophages (BMDMs) and dendritic cells (BMDCs) were isolated and differentiated from bone marrow cells of 5-week-old male BALB/C mice. Briefly, bone marrow cells from mice were differentiated into BMDMs or BMDCs by incubating for 7 days in the presence of macrophage colony-stimulating factor (M-CSF, 20 ng/mL), or interleukin-4 (IL-4, 20 ng/mL), granulocyte macrophage colony-stimulating factor (GM-CSF, 20 ng/mL) and 0.1% β-mercaptoethanol, respectively. M1 macrophages were prepared by incubating M0 macrophages with LPS (1 µg/ml) for 5 h. CD8^+^ T cells were collected from BALB/c mice using a CD8^+^ T cell enrichment column, followed by activation for 5 days via a T cell activation/expansion kit (cat# 130-093-627, Miltenyi Biotechnology (Bergisch Gladbach, Germany). The morphology of collected immune cells was observed using an optical microscopy. After 24 h of stabilization, the cells were solubilized with lysis buffer containing 1% protease inhibitor at 4 °C, and each lysate was centrifuged at 8000 rpm for 20 min. Proteins in supernatants were quantified via BCA protein quantification kit and resolved under sodium dodecyl sulfate–polyacrylamide gel electrophoresis on 12% gels, which were transferred onto polyvinylidene difluoride (PVDF) membranes. Then, membranes were incubated with tris buffered saline with tween 20 (TBS-T) containing 5% bovine serum albumin (BSA) to block non-specific IgG binding and further incubated with rabbit anti-mouse cathepsin B antibody at 4 °C for 24 h. Finally, membranes were incubated with horseradish peroxidase (HRP)-conjugated mouse anti-rabbit antibody for 90 min. Immunoreactive bands were detected using an enhanced chemiluminescence system.

### Cellular uptake of Aposomes

To assess the cellular uptake of Aposomes in cultured cells, 2 × 10^6^ CT26 and 4T1 cancer cells, H9C2 and HDF normal cells, and immune cells of macrophages, DCs and T cells were seeded in 35 mm glass-bottom cell culture dishes. After 24 h of stabilization, each cell was incubated with an equivalent DOX dose (2 μM) of Aposomes or DOXIL at 37 °C for 48 h. The cells were then washed with DPBS, fixed with 4% paraformaldehyde for 10 min, and stained with 4ʹ,6-diamidino-2-phenylindole (DAPI) for 5 min. For fluorescence imaging of suspension T cells, they were mounted using cover-glass with PBS in 35 mm glass-bottom cell culture dishes. Then, cellular uptake of Aposomes and DOXIL was observed by using confocal laser-scanning microscope (CLSM, Leica TCS SP8, Leica Microsystems GmbH). The fluorescence intensity of Aposomes and DOXIL within the cells was analyzed using Image-Pro Plus software (Media Cybernetic, Rockville, MD, USA). The cellular uptake of each drug in different cell lines were quantified using flow cytometer (CytoFLEX, BECKMAN COULTER, USA).

### Cytotoxicity study

The cytotoxicity of Aposomes and DOXIL was assessed in CT26 and 4T1 cancer cells, H9C2 and HDF normal cells, and immune cells of macrophages, DCs and T cells. Briefly, 5 × 10^3^ cells were seeded in 96-well cell culture plates and cultured at 37  C for 24 h. Then, different concentrations (0.001–10 µM based on DOX contents) of Aposomes or DOXIL were incubated with each cell for 48 h. Finally, 20 μl Cell Counting Kit-8 (CCK-8) solution was added and then further incubated for 20 min. The cell viability was measured using a UV/Vis microplate reader.

### DAMPs analysis

To assess the DAMPs expression owing to an ICD effect by synergistic activity of SMAC-P and DOX of Aposomes, 1 × 10^5^ CT26 cells were seeded in 35-mm glass-bottom cell culture dishes to which Aposomes or DOXIL was added at an equivalent DOX concentration of 2 µM. After 48 h of incubation, the cells were stained with Alexa Fluor 647 fluorescent dye-conjugated anti-calreticulin (CRT) antibodies at 4 °C for 1 h, and cell culture medium were also collected to assess the HMGB1 and ATP from the cancer cells. After staining with CRT antibodies, cells were washed twice with DPBS, fixed with 4% paraformaldehyde for 10 min, and stained with DAPI for 5 min. Surface calreticulin expression within the CT26 cells treated with Aposomes or DOXIL was observed using CLSM. Meanwhile, HMGB1 and ATP released from Aposome- or DOXIL-treated CT26 cells to the cell culture medium were quantitatively measured via western blot and commercialized ATP assay kit, respectively.

### Co-culture assays

The effective ICD effects by Aposomes were evaluated through several co-culture assays. First, phagocytosis enhancement of macrophages by DAMPs from cancer cells treated with Aposomes was assessed. For these analyses, bone marrow cells were collected from 5-week-old male BALB/C mice and cultured with differentiation media containing M-CSF (20 ng/mL) for 7 days, followed by labeling with CellTracker Green. Meanwhile, CT26 cells were treated with Aposomes or DOXIL (2 μM based on DOX contents) at 37  C for 48 h and further labeled with a pH-sensitive dye, pHrodo-succinimidyl ester. Then, both cells were co-cultured at 37  C for 2 h, and the phagocytosis of cancer cells was observed using a CLSM. To assess the DC maturation and T cell activation according to ICD effects by Aposomes, 1 × 10^6^ CT26 cells were cultured in 100-pi cell culture dishes and treated with Aposomes or DOXIL (2 μM based on DOX contents) at 37  C for 48 h. Then, CT26 cells after each treatment were co-cultured with lymphocytes from 5 week-old male BALB/C mice at 37  C for 2 h, and the population of CD11c^+^CD40^+^CD86^+^ mature DCs and CD45^+^CD3^+^CD8^+^ activated T cells within the lymphocytes were analyzed via a flow cytometer (CytoFLEX, BECKMAN COULTER, USA).

### Biodistribution of Aposomes in colon tumor models

All experiments with animals were performed in compliance with the relevant guidelines according to Institutional Animal Care and Use Committee (IACUC) of Korea Institute of Science and Technology (KIST; Approved number: 2023–013). Pharmacokinetics (PK) properties were assessed in the BALB/c mice after I.V. injection with an equivalent dose of 3 mg/kg DOX content of free DOX, SMAC-P-FRRG-DOX or Aposomes. At the pre-determined time after treatment, blood were collected from the mice via cardiac puncture after deep anesthesia, followed by analysis of DOX fluorescence with fluorescence detector. The PK parameters, such as area under the curves (AUC), clearance (CL) and C_max_ were calculated using a WinNonlin software. The tumor targeting of Aposomes was assessed in CT26 colon and 4T1 breast tumor-bearing mice. For these analyses, 1 × 10^6^ CT26 cells or 4T1 cells were subcutaneously injected into the flank of mice. When the tumor volumes were approximately 200 ± 10 mm^3^, each tumor-bearing mouse were intravenously injected with an equivalent 3 mg/kg DOX dose of Aposome, DOXIL or free DOX. Then, whole-body near-infrared fluorescence (NIRF) imaging was performed using an in vivo imaging system (IVIS^®^ Lumina Series III, PerkinElmer, USA). Fluorescence intensities of Aposome, DOXIL or free DOX within the tumor regions was quantified using a Living Image^®^ software (PerkinElmer, USA). For ex vivo fluorescence imaging, CT26 colon tumor-bearing mice were sacrificed to collect tumor and major organ (liver, lung, spleen, and heart) tissues. Fluorescence intensities of Aposome, DOXIL or free DOX in collected tissues from mice were analyzed using an in vivo imaging system. For histological analysis, collected tumor tissues were cut into 8 μm thick sections and stained with APC fluorescent dye-conjugated anti-CD31 antibody at 4  C for 1 h. Then, slide-mounted tumor sections were stained with DAPI for 10 min, after which DOX fluorescence of Aposome, DOXIL or free DOX in tumor tissues from mice was observed using a CLSM.

### In vivo* antitumor efficacy and immune response of Aposomes*

The antitumor efficacy and immune response of Aposomes were assessed in CT26 colon and 4T1 breast tumor-bearing mice. First, the mice were randomly divided into four groups of (i) saline, (ii) free DOX, (iii) DOXIL and (iv) Aposomes. Then, an equivalent 3 mg/kg DOX dose of Aposomes, DOXIL and free DOX was injected into mice once every 3 days, at which time tumor volumes were approximately 50 ± 30 mm^3^. Antitumor efficacy was assessed by measuring tumor volumes that are calculated as the largest diameter × the smallest diameter^2^ × 0.53. To analyze the increase of tumor-infiltrating lymphocytes by antitumor immune response, tumor tissues were collected from mice on day 13 after treatment, and single cells were isolated from tumor tissues using a tumor dissociation kit. Following single cell counting, the cells were incubated with FcBlock for 10 min to avoid non-specific antibody binding. Then, multi-parameter staining was performed at 4 °C for 50 min to identify the following populations within the tumor tissues: (i) CRT-positive cancer cells (CD45^−^CRT^+^), (ii) mature DCs (CD11c^+^CD40^+^CD86^+^), (iii) cytotoxic T cells (CD45^+^CD3^+^CD8^+^) and (iv) regulatory T lymphocytes (Tregs; CD45^+^CD3^+^CD4^+^CD25^+^). To assess whether Aposomes potentiate the ICB therapy, anti-PD-L1 antibody (αPD-L1 Ab, 10 mg/kg) was simultaneously injected via tail vein once every three days. On day 100 after treatment, the establishment of immunologic memory in mice that experienced complete tumor regression by Aposomes plus αPD-L1 Ab was rechallenged with 1 × 10^6^ CT26 cells via intravenous injection. Rechallenged tumor volumes were measured once every two days, and the population of splenic effector/memory CD8^+^ T cells (CD8^+^CD44^+^CD62L^low^) were analyzed using a flow cytometry on day 120 after treatment (on day 20 after tumor rechallenge).

### Statistical Analysis

Statistical significance between two groups was analyzed using Student’s t test. In case of more than two groups, one-way analysis of variance (ANOVA) was used, and multiple comparisons were performed using Tukey–Kramer post-hoc test. Survival results were plotted using Kaplan − Meier curves and analyzed using the log-rank test. All results are presented as mean ± SD, and P values of < 0.05*, < 0.01** and < 0.001*** were considered statistically significant.

### Supplementary Information

**Additional file 1: Figure S1.** Synthetic route to prepare the cancer-specific and pro-apoptotic prodrug, SMAC-P-FRRG-DOX. **Figure S2.** ^1^H-NMR spectra of SMAC-P-FRRG-DOX in DMSO-*d*_6_ (1 mg/ml). **Figure S3.** Zeta potential of SMAC-P-FRRG-DOX and Aposomes in D.W (1 mg/ml), as confirmed using a Zetasizer. **Figure S4.** **a** Size distribution of SMAC-P-FRRG-DOX in saline (1 mg/kg). **b** Particle stability of SMAC-P-FRRG-DOX nanoparticles in mouse plasma. **Figure S5.** **a** Metabolite assay of SMAC-P-FRRG-DOX after incubation with cathepsin B enzyme. **b** Cleavage behavior of SMAC-P-FRRG-DOX after 24 h of incubation in MES buffer containing cathepsin B. **Figure S6.** Cathepsin B expression levels in CT26 and 4T1 cancer cells, H9C2 and HDF normal cells, and immune cells of M0 and M1 macrophages, dendritic cells (DCs) and T cells. **Figure S7.** Relative DOX fluorescence in cytosol or nucleus of CT26 after treatment with free DOX, DOXIL or Aposomes for 48 h with or without cathepsin B inhibitor, Z-FA-FMK. **Figure S8.** Relative DOX fluorescence in cytosol or nucleus of CT26 and 4T1 cancer cells, H9C2 and HDF normal cells, and immune cells of M0 and M1 macrophages, DCs and T cells after treatment with (**a**) free DOX or (**b**) DOXIL for 48 h. **Figure S9.** **a** The morphology of immune cells of M0 and M1 macrophages, dendritic cells (DCs) and T cells, as confirmed by optical microscope. **b**, **c** Fluorescence images of CT26 and 4T1 cancer cells, H9C2 and HDF normal cells, and immune cells of macrophages, DCs and T cells, which are treated with free DOX, DOXIL or Aposomes for 48 h. **Figure S10.** IC50 values of free DOX, DOXIL and Aposomes in CT26 and 4T1 cancer cells, H9C2 and HDF normal cells, and immune cells of M0 and M1 macrophages, dendritic cells (DCs) and T cells after 48 h of treatment. **Figure S11.** Expression levels of IAP in CT26 cells after treatment with free DOX, DOXIL or Aposomes for 48 h. **Figure S12.** HMGB1 released from CT26 cells treated with free DOX, DOXIL or Aposomes (2 µM based on DOX contents) for 48 h. **Figure S13**. **a** Pharmacokinetic (PK) properties of DOX, SMAC-P-FRRG-DOX and Aposome in BALB/c mice after intravenous injection with an equivalent dose of 3 mg/kg based on DOX content. **b** The PK parameters, area under the curves (AUC), clearance (CL) and C_max_, were determined using a WinNonlin software. **Figure S14.** Quantitative analyses for DOX fluorescence in tumor tissues from CT26 colon tumor-bearing mice after treatment with saline or an equivalent DOX dose (3 mg/kg) of free DOX, DOXIL or Aposomes for 24 h. **Figure S15.** Tumor targeting of Aposomes in breast tumor models. **Figure S16.** **a** Individual tumor growth curves of CT26 colon tumor-bearing mice during treatment with an equivalent 3 mg/kg DOX dose of free DOX, DOXIL or Aposomes once every three days. **b **Optical images of tumor tissues on day 13 after treatment. **Figure S17.** Relative expression levels of IAP in cancer cells after 13 days of treatment with free DOX, DOXIL or Aposomes, confirmed as western blot analysis. **Figure S18.** **a** Major organs (liver, lung, kidney and heart) stained with H and E on day 13 after treatment. **b** Body weight changes of mice during treatment with an equivalent 3 mg/kg DOX dose of free DOX, DOXIL or Aposomes once every 3 days. **Figure S19.** **a** Individual tumor growth curves of 4T1 breast tumor-bearing mice during treatment with an equivalent 3 mg/kg DOX dose of free DOX, DOXIL, Aposomes or Aposomes plus αPD-L1 Ab once every three days. **b** Optical images of tumor tissues on day 13 after treatment. **c** Tumor tissues stained with anti-IAPs antibody or TUNEL on day 13 after treatment. **d** Mice survival during treatment. **e** Various immune cells population within tumor tissues on day 13 after treatment.

## Data Availability

All relevant data are available with the article and its Supporting Information files or available from the corresponding authors upon reasonable request.
