# Supplementary material for: Cancer cell-specific and pro-apoptotic SMAC peptide-doxorubicin conjugated prodrug encapsulated aposomes for synergistic cancer immunotherapy
Source: J Nanobiotechnology. 2024 Mar 13;22:109. doi: 10.1186/s12951-024-02314-w (PMC10938764; doi:10.1186/s12951-024-02314-w)
Supplement: Supplementary file 1 — Additional file 1: Figure S1. Synthetic route to prepare the cancer-specific and pro-apoptotic prodrug, SMAC-P-FRRG-DOX. Figure S2. 1H-NMR spectra of SMAC-P-FRRG-DOX in DMSO-d6 (1 mg/ml). Figure S3. Zeta potential of SMAC-P-FRRG-DOX and Aposomes in D.W (1 mg/ml), as confirmed using a Zetasizer. Figure S4. a Size distribution of SMAC-P-FRRG-DOX in saline (1 mg/kg). b Particle stability of SMAC-P-FRRG-DOX nanoparticles in mouse plasma. Figure S5. a Metabolite assay of SMAC-P-FRRG-DOX after incubation with cathepsin B enzyme. b Cleavage behavior of SMAC-P-FRRG-DOX after 24 h of incubation in MES buffer containing cathepsin B. Figure S6. Cathepsin B expression levels in CT26 and 4T1 cancer cells, H9C2 and HDF normal cells, and immune cells of M0 and M1 macrophages, dendritic cells (DCs) and T cells. Figure S7. Relative DOX fluorescence in cytosol or nucleus of CT26 after treatment with free DOX, DOXIL or Aposomes for 48 h with or without cathepsin B inhibitor, Z-FA-FMK. Figure S8. Relative DOX fluorescence in cytosol or nucleus of CT26 and 4T1 cancer cells, H9C2 and HDF normal cells, and immune cells of M0 and M1 macrophages, DCs and T cells after treatment with (a) free DOX or (b) DOXIL for 48 h. Figure S9. a The morphology of immune cells of M0 and M1 macrophages, dendritic cells (DCs) and T cells, as confirmed by optical microscope. b, c Fluorescence images of CT26 and 4T1 cancer cells, H9C2 and HDF normal cells, and immune cells of macrophages, DCs and T cells, which are treated with free DOX, DOXIL or Aposomes for 48 h. Figure S10. IC50 values of free DOX, DOXIL and Aposomes in CT26 and 4T1 cancer cells, H9C2 and HDF normal cells, and immune cells of M0 and M1 macrophages, dendritic cells (DCs) and T cells after 48 h of treatment. Figure S11. Expression levels of IAP in CT26 cells after treatment with free DOX, DOXIL or Aposomes for 48 h. Figure S12. HMGB1 released from CT26 cells treated with free DOX, DOXIL or Aposomes (2 µM based on DOX contents) for 48 h. Figur [file 12951_2024_2314_MOESM1_ESM.docx]

**Addition file Information for**

Cancer Cell-Specific and Pro-Apoptotic SMAC Peptide-Doxorubicin Conjugated Prodrug Encapsulated Aposomes for Synergistic Cancer Immunotherapy

Jinseong Kim^1,2,#^, Man Kyu Shim^3,#^, Yujeong Moon^3,4^, Jeongrae Kim^1,2^, Hanhee Cho^1^, Wan Su Yun^1,2^, Nayeon Shim^1^, Joon-Kyung Seong^4^, Younghyun Lee^1^, Dong-Kwon Lim^2^, Kwangmeyung Kim^1,^*

^1^College of Pharmacy, Graduate School of Pharmaceutical Sciences, Ewha Womans University, Seoul 03760, Republic of Korea.

^2^KU-KIST Graduate School of Converging Science and Technology, Korea University, Seoul, 02841, Republic of Korea.

^3^Medicinal Materials Research Center, Biomedical Research Division, Korea Institute of Science and Technology (KIST), Seoul, 02792, Republic of Korea.

^4^Department of Bioengineering, Korea University, Seoul, 02841, Republic of Korea.

^#^These authors contributed equally to this work.

*Correspondence and requests for materials should be addressed to **K. Kim** (E-mail: kimkm@ewha.ac.kr; address: College of Pharmacy, Graduate School of Pharmaceutical Sciences, Ewha Womans University, Seoul 03760, Republic of Korea.).

**
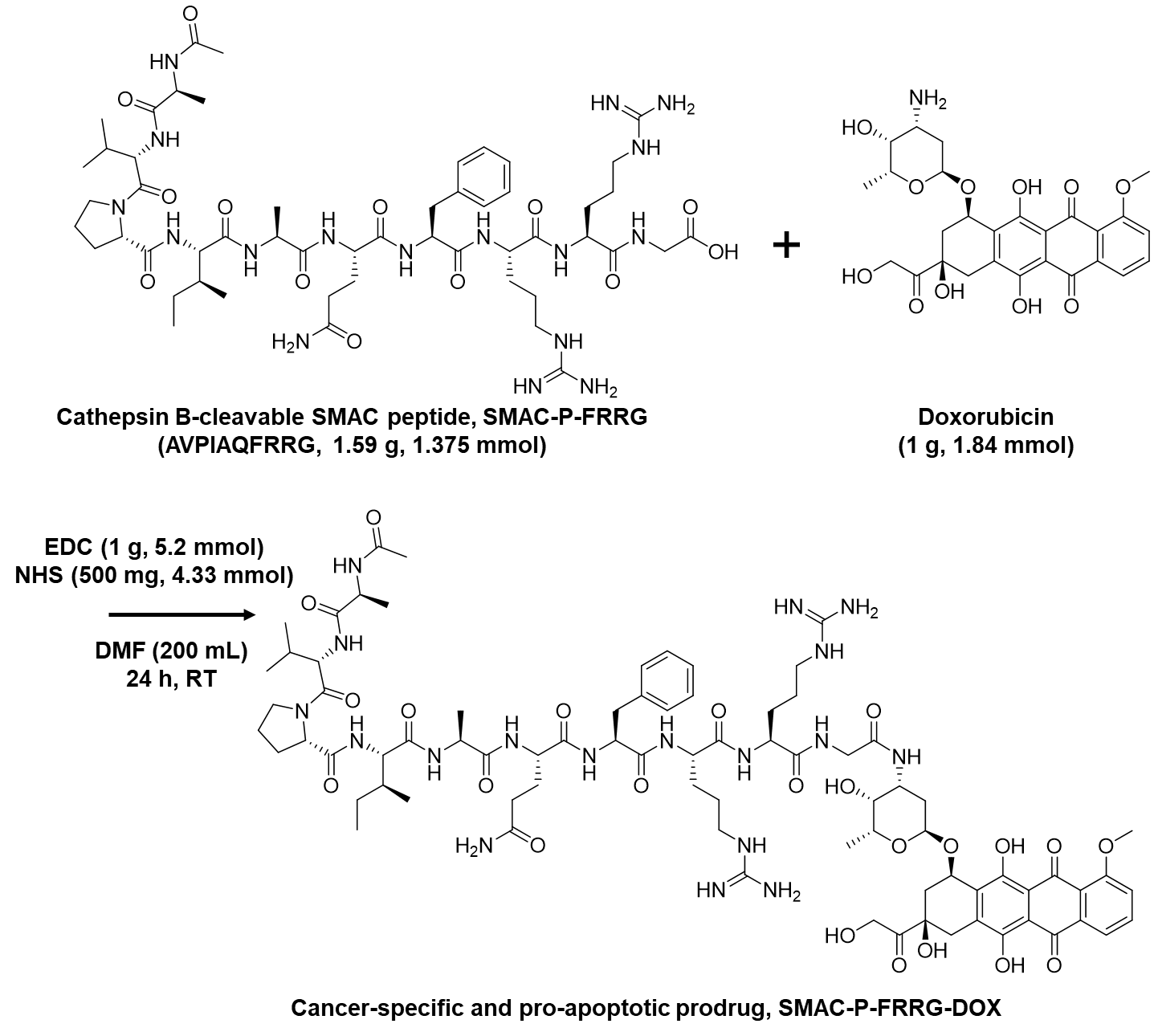
**

**Figure S1.** Synthetic route to prepare the cancer-specific and pro-apoptotic prodrug, SMAC-P-FRRG-DOX.


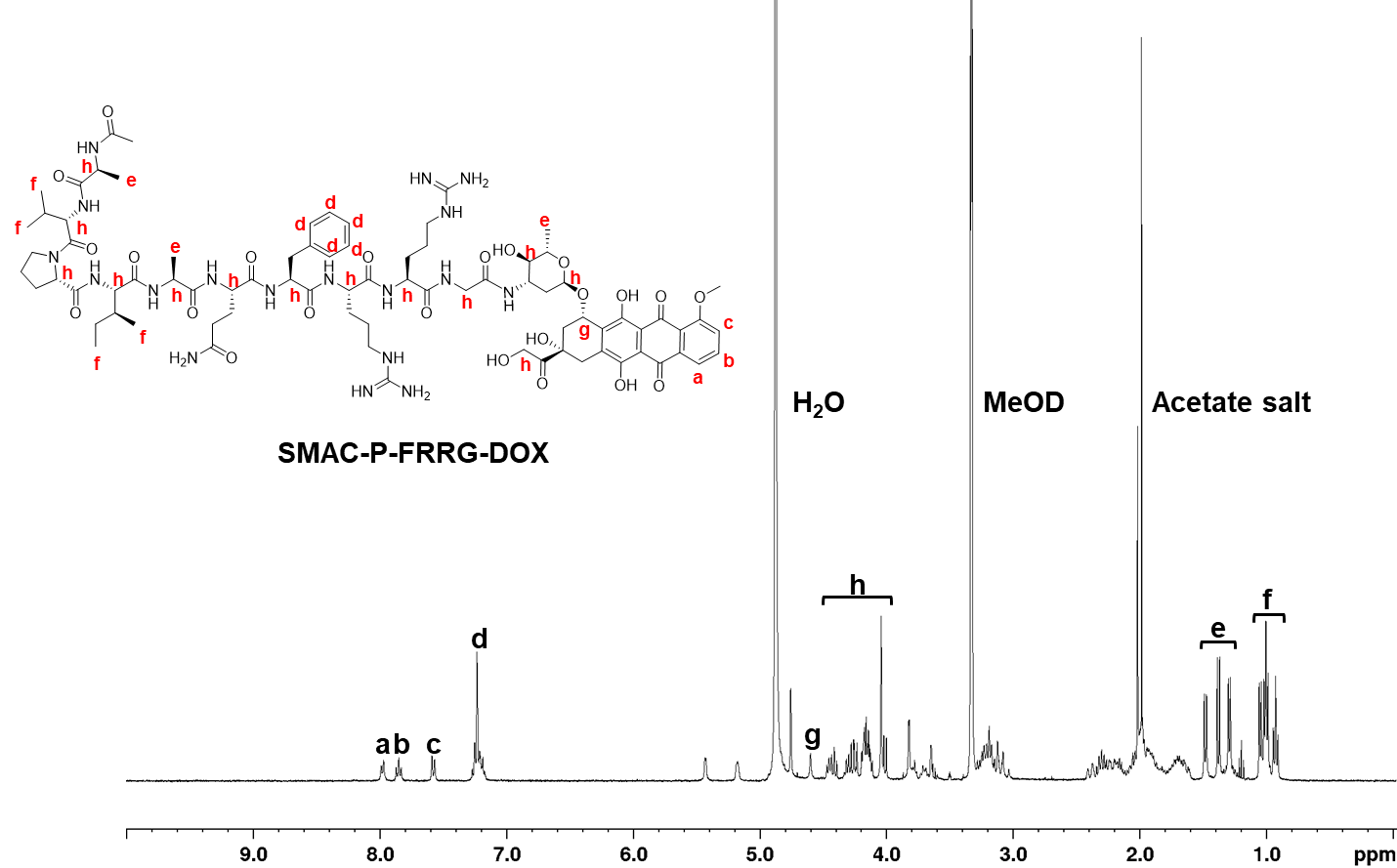


**Figure S2.** ^1^H-NMR spectra of SMAC-P-FRRG-DOX in DMSO-*d*_6_ (1 mg/ml).


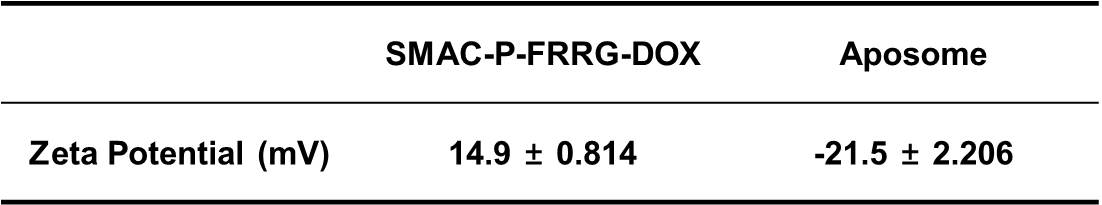


**Figure S3.** Zeta potential of SMAC-P-FRRG-DOX and Aposomes in D.W (1 mg/ml), as confirmed using a Zetasizer.


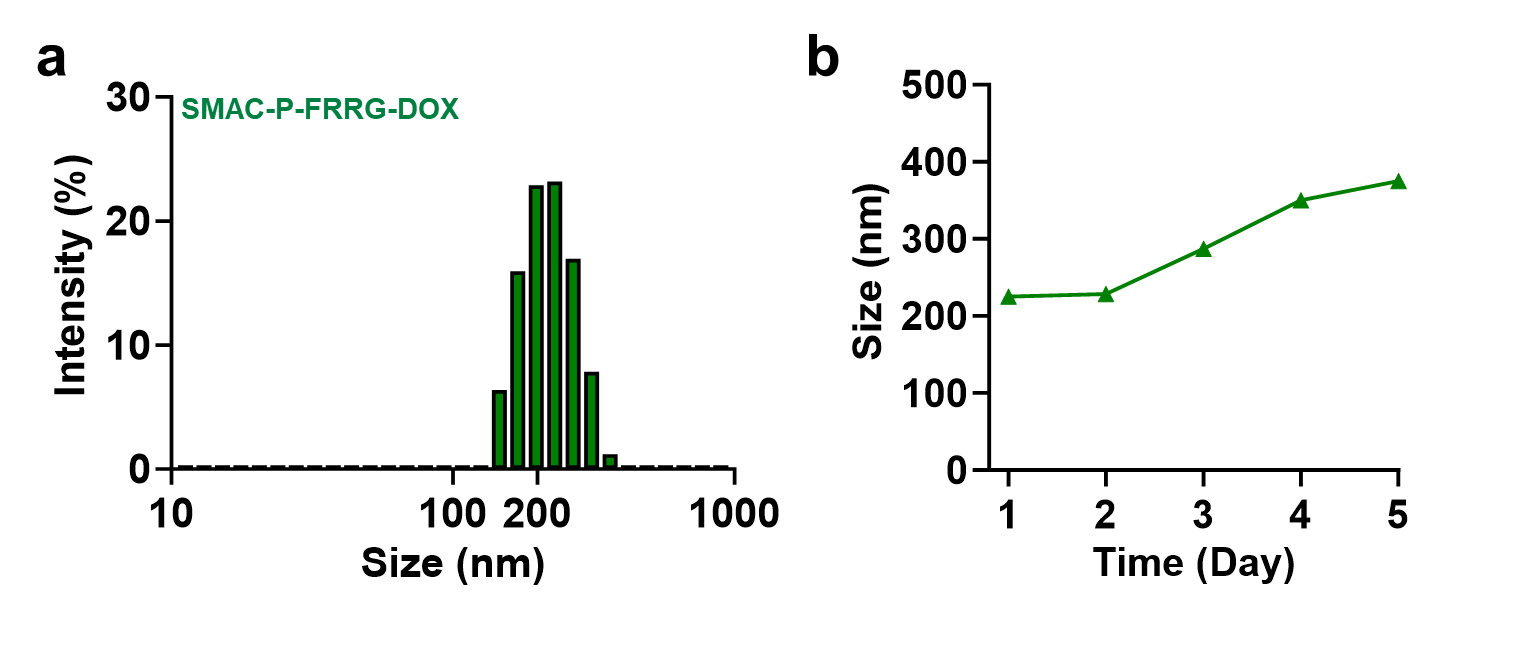


**Figure S4. (a)** Size distribution of SMAC-P-FRRG-DOX in saline (1 mg/kg). **(b)** Particle stability of SMAC-P-FRRG-DOX nanoparticles in mouse plasma.


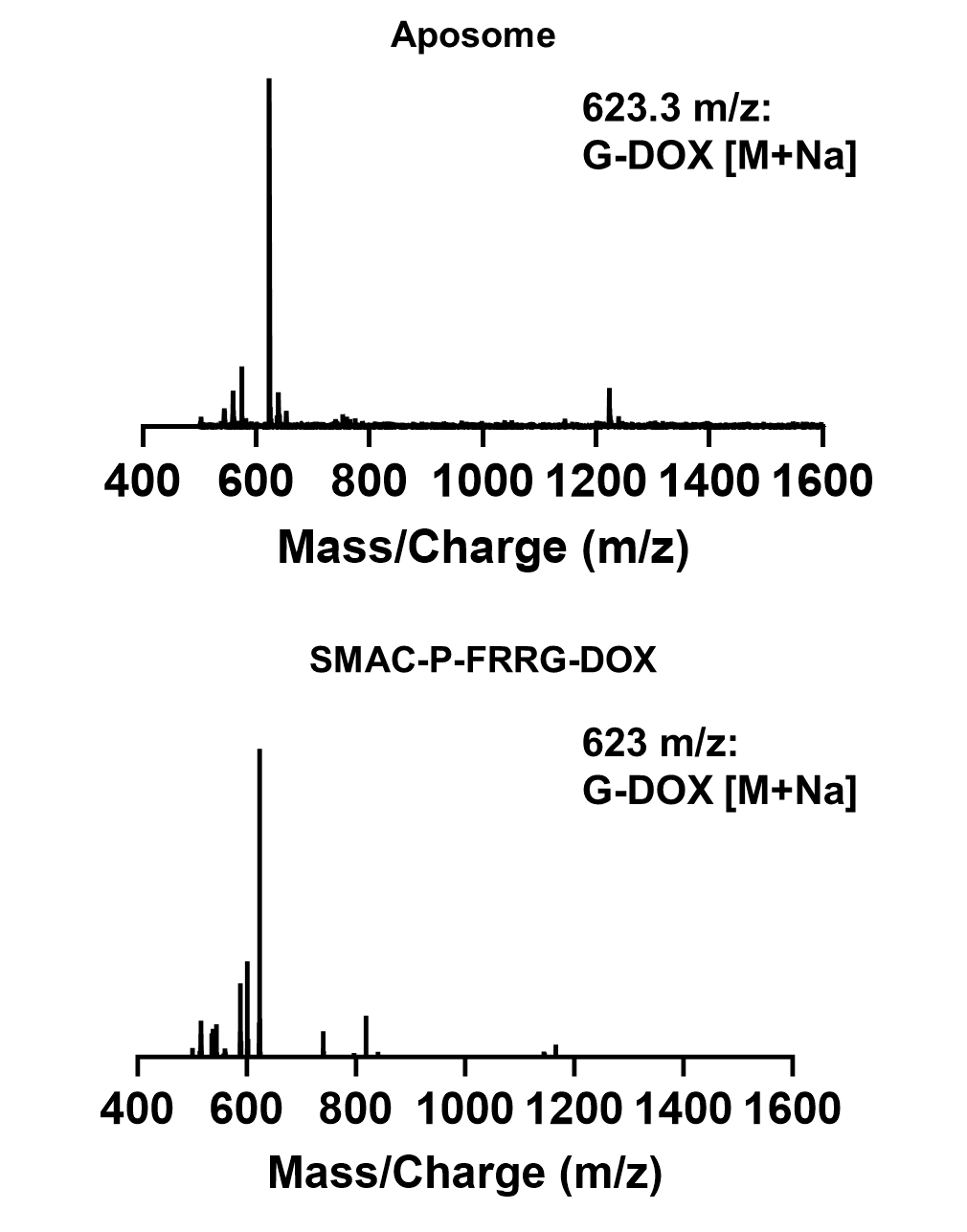


**Figure S5.** **(a)** Metabolite assay of SMAC-P-FRRG-DOX after incubation with cathepsin B enzyme. **(b)** Cleavage behavior of SMAC-P-FRRG-DOX after 24 h of incubation in MES buffer containing cathepsin B.


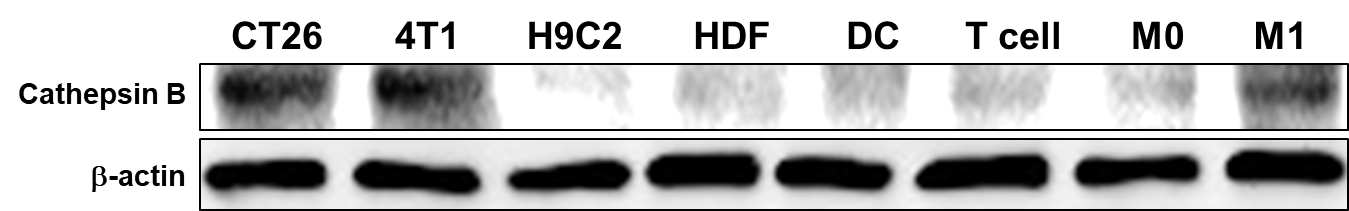


**Figure S6.** Cathepsin B expression levels in CT26 and 4T1 cancer cells, H9C2 and HDF normal cells, and immune cells of M0 and M1 macrophages, dendritic cells (DCs) and T cells.

**Figure S7.** Relative DOX fluorescence in cytosol or nucleus of CT26 after treatment with free DOX, DOXIL or Aposomes for 48 h with or without cathepsin B inhibitor, Z-FA-FMK.


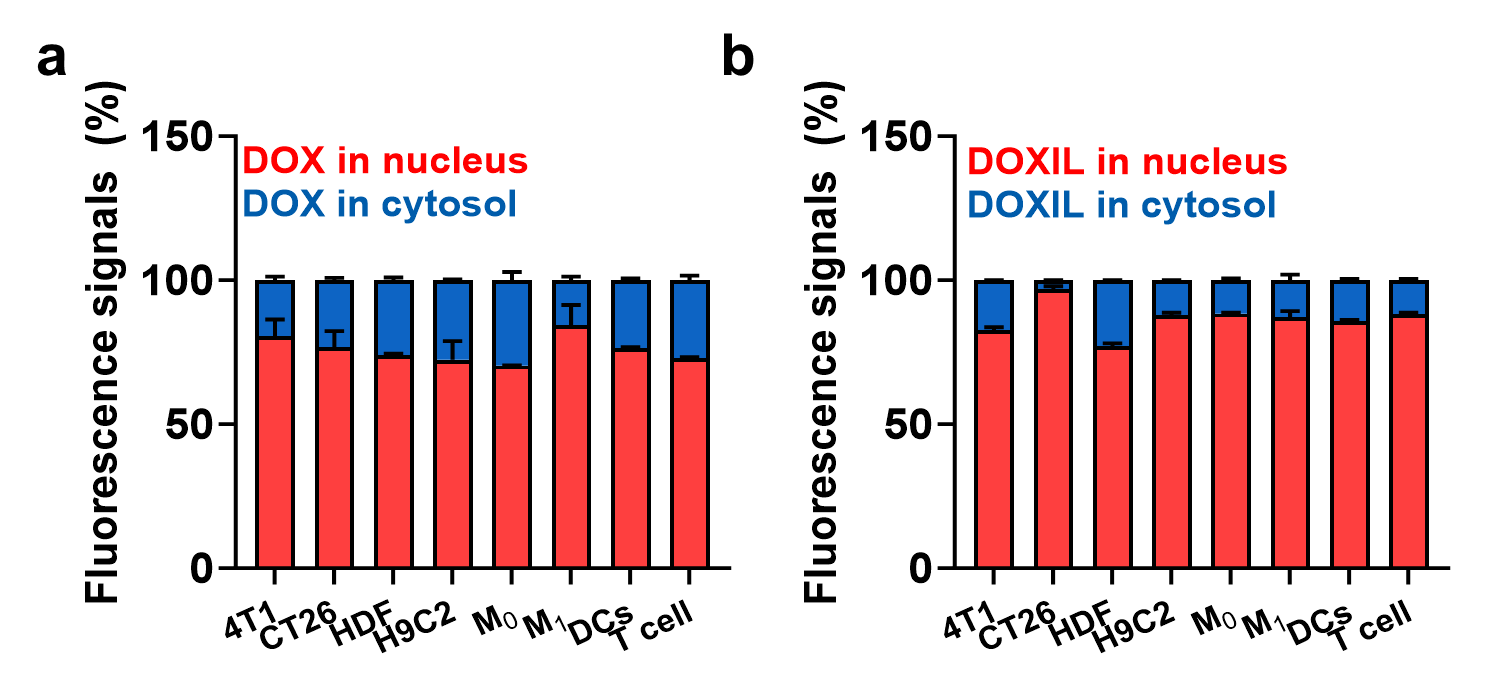


**Figure S8.** Relative DOX fluorescence in cytosol or nucleus of CT26 and 4T1 cancer cells, H9C2 and HDF normal cells, and immune cells of M0 and M1 macrophages, DCs and T cells after treatment with (a) free DOX or (b) DOXIL for 48 h.


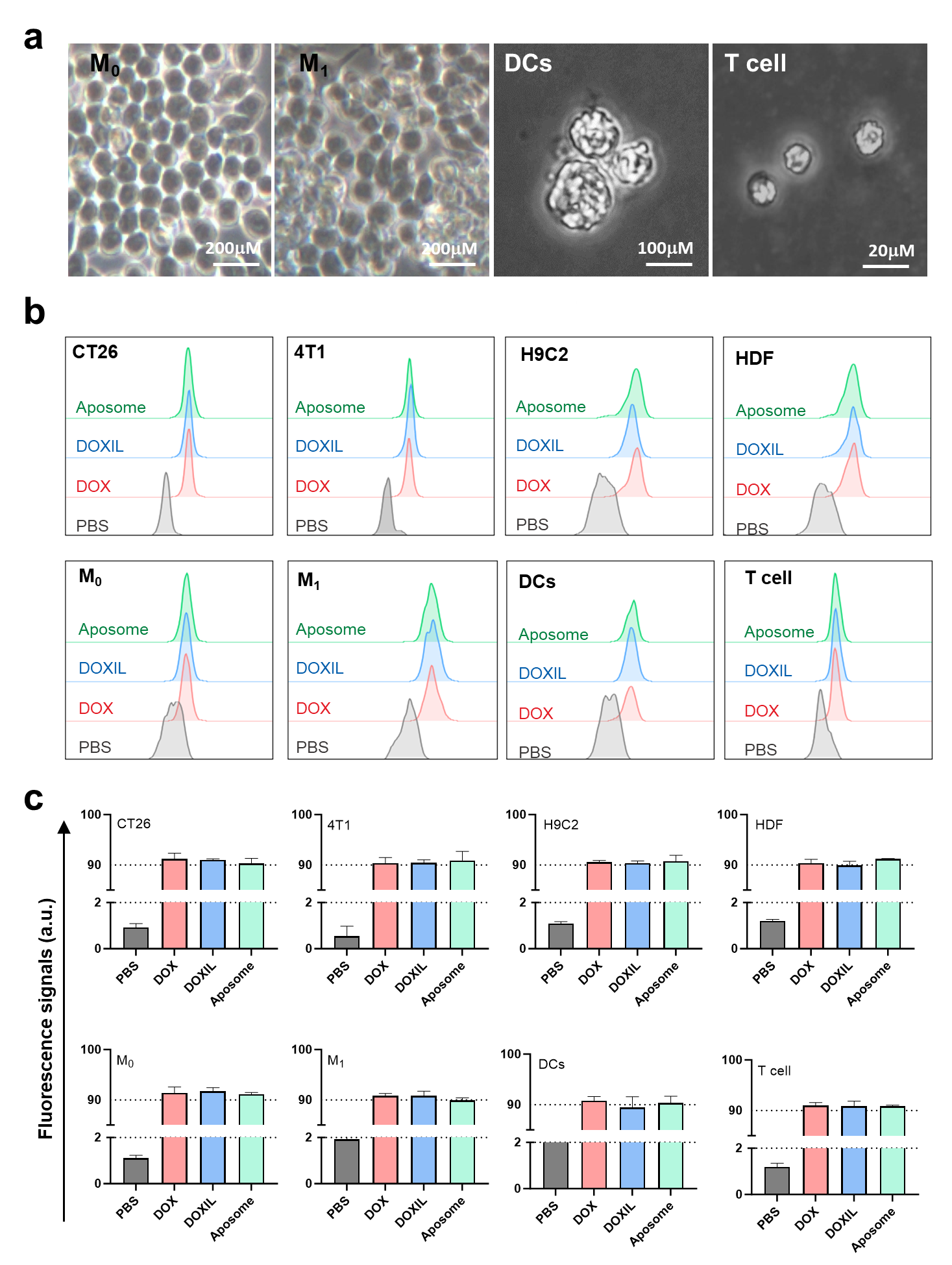


**Figure S9.** **(a)** The morphology of immune cells of M0 and M1 macrophages, dendritic cells (DCs) and T cells, as confirmed by optical microscope. **(b, c)** Fluorescence images of CT26 and 4T1 cancer cells, H9C2 and HDF normal cells, and immune cells of macrophages, DCs and T cells, which are treated with free DOX, DOXIL or Aposomes for 48 h.


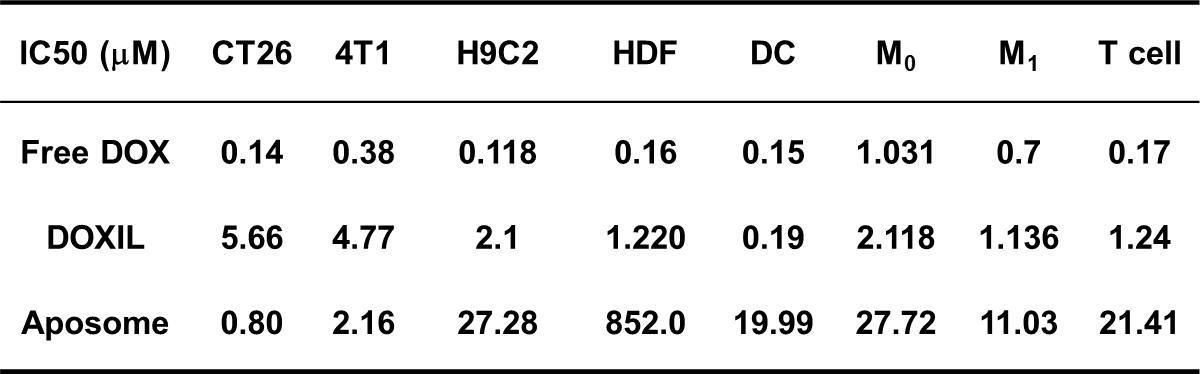


**Figure S10.** IC50 values of free DOX, DOXIL and Aposomes in CT26 and 4T1 cancer cells, H9C2 and HDF normal cells, and immune cells of **M0 and M1 macrophages**, dendritic cells (DCs) and T cells after 48 h of treatment.


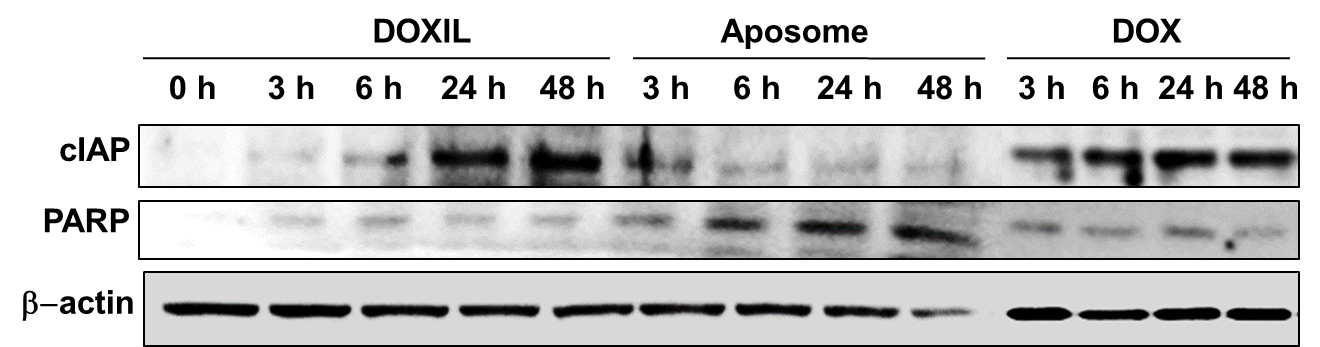


**Figure S11.** Expression levels of IAP in CT26 cells after treatment with free DOX, DOXIL or Aposomes for 48 h.


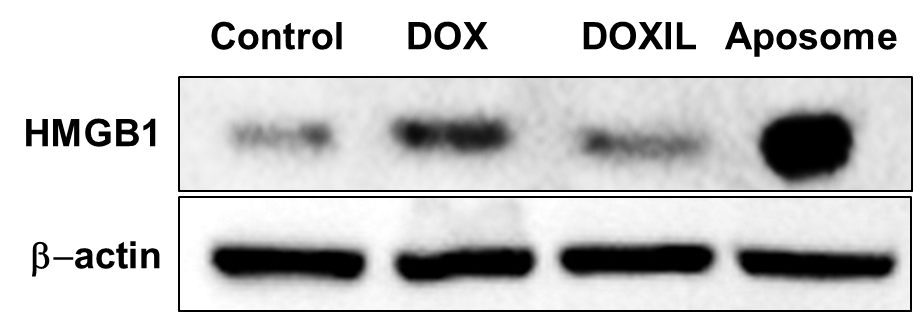


**Figure S12.** HMGB1 released from CT26 cells treated with free DOX, DOXIL or Aposomes (2 μM based on DOX contents) for 48 h.


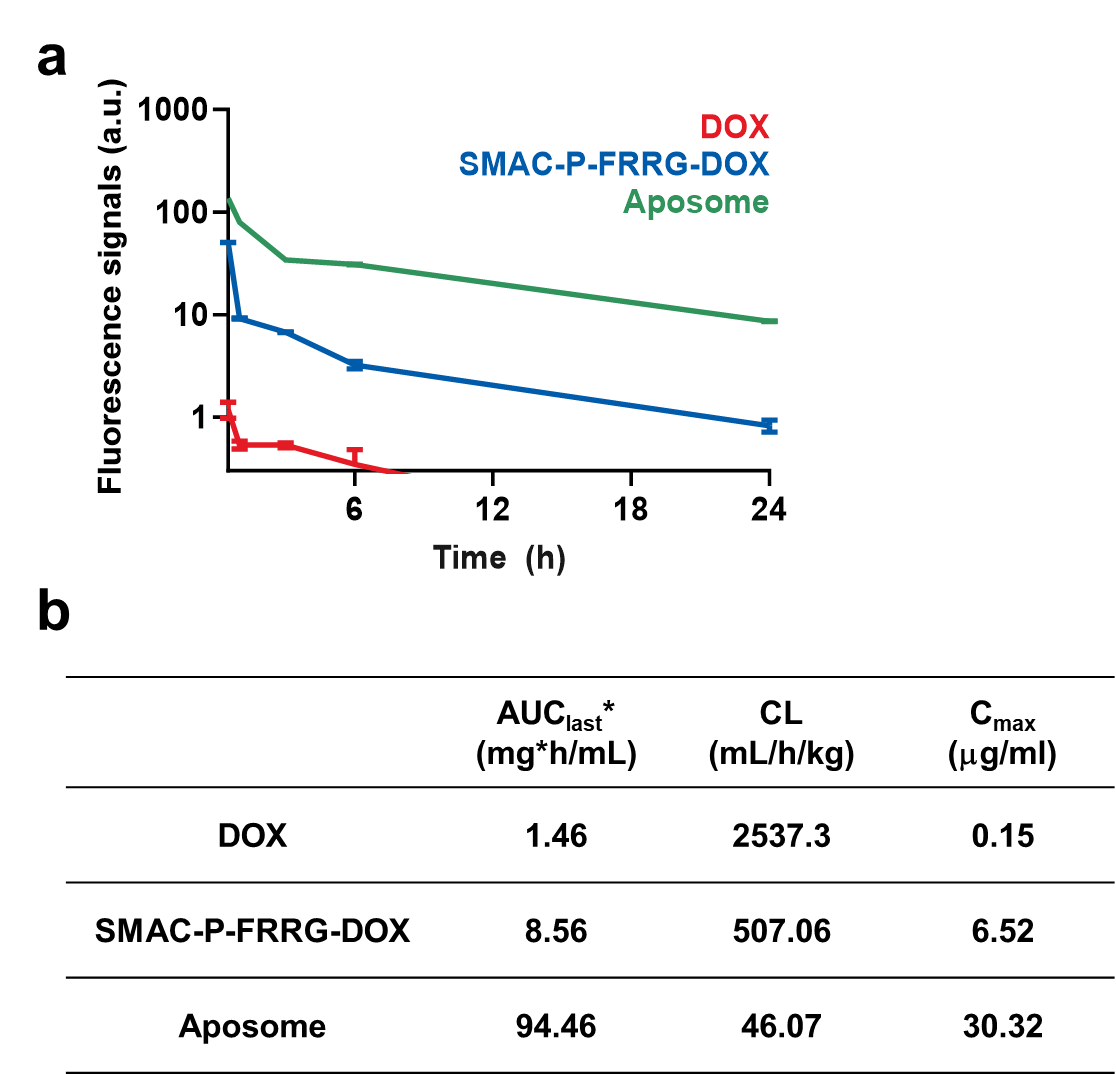


**Figure S13.** **(a)** Pharmacokinetic (PK) properties of DOX, SMAC-P-FRRG-DOX and Aposome in BALB/c mice after intravenous injection with an equivalent dose of 3 mg/kg based on DOX content. **(b)** The PK parameters, area under the curves (AUC), clearance (CL) and C_max_, were determined using a WinNonlin software.

**Figure S14.** Quantitative analyses for DOX fluorescence in tumor tissues from CT26 colon tumor-bearing mice after treatment with saline or an equivalent DOX dose (3 mg/kg) of free DOX, DOXIL or Aposomes for 24 h.


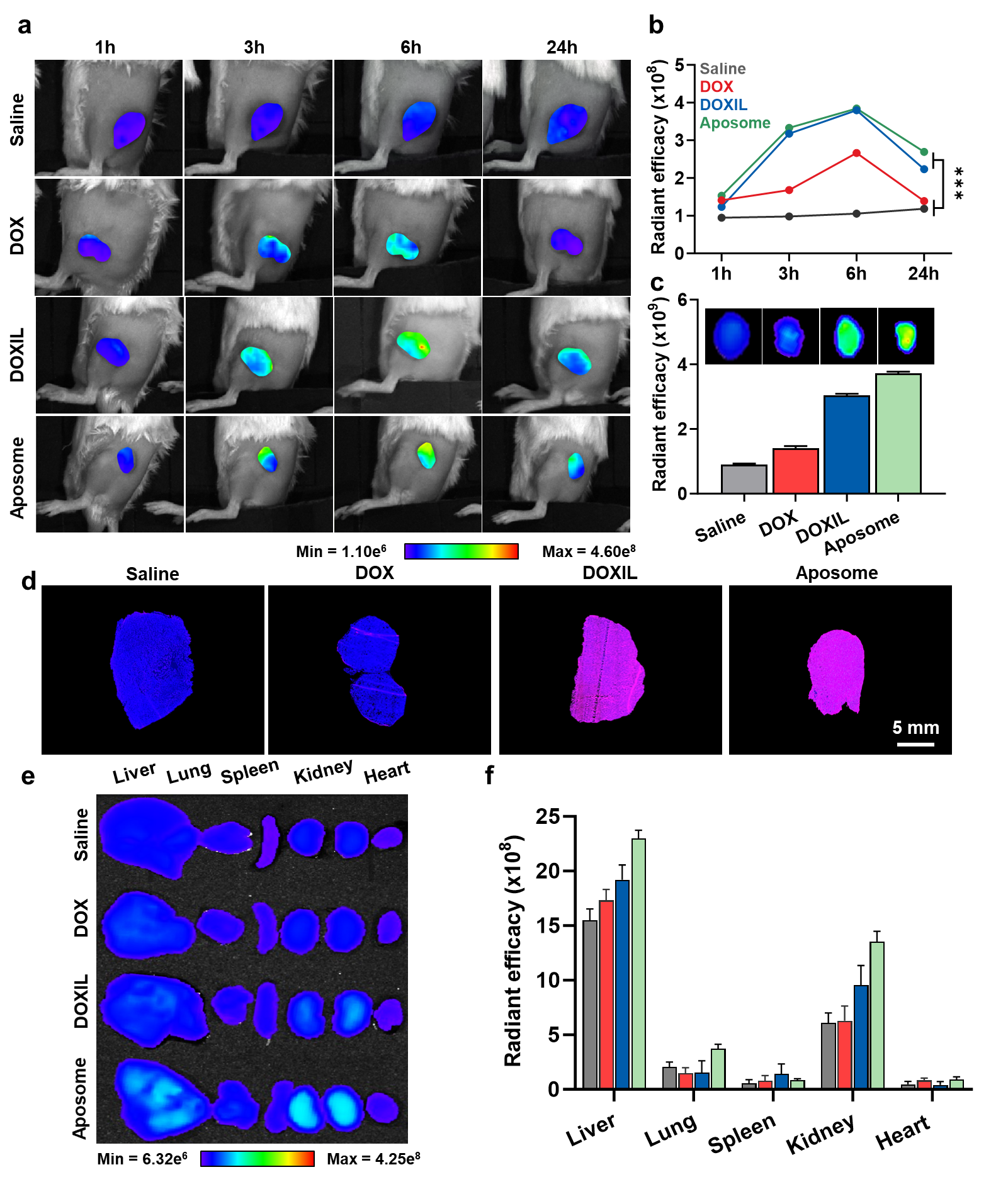


**Figure S15. Tumor targeting of Aposomes in breast tumor models. (a)** NIRF images of 4T1 breast tumor-bearing mice after treatment with saline or an equivalent DOX dose (3 mg/kg) of free DOX, DOXIL or Aposomes. **(b)** Quantitative analyses for DOX fluorescence signals within the tumor regions. **(c)** Fluorescence images and quantitative analyses of exercised tumor tisseus after 24 h of treatment. **(d)** Histological analyses of tumor tissues from CT26 colon tumor-bearing mice after 24 h of treatment. **(e, f)** *Ex vivo* fluorescence images of CT26 colon tumor-bearing mice after 24 h of treatment.


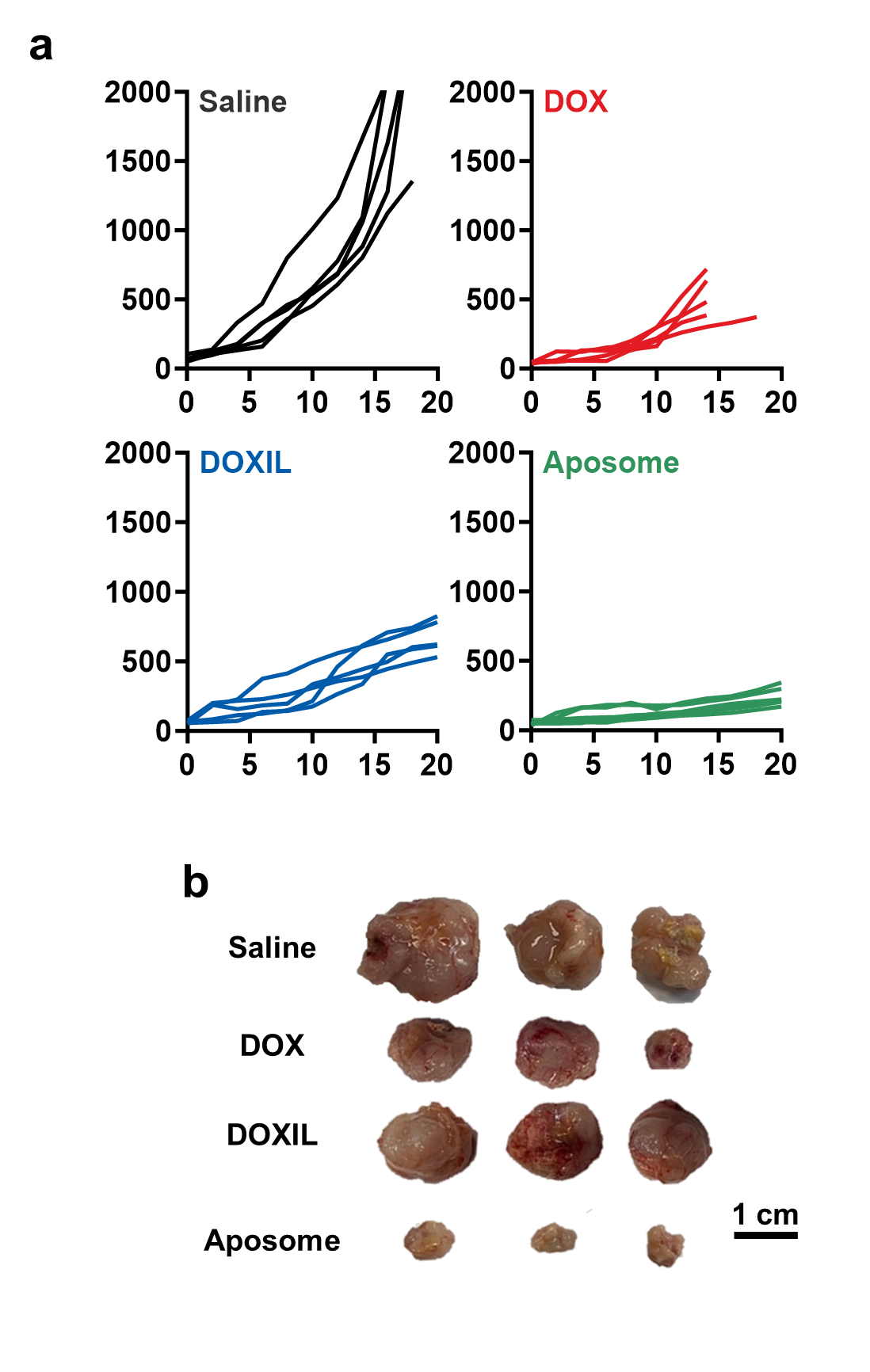


**Figure S16.** **(a)** Individual tumor growth curves of CT26 colon tumor-bearing mice during treatment with an equivalent 3 mg/kg DOX dose of free DOX, DOXIL or Aposomes once every three days. **(b)** Optical images of tumor tissues on day 13 after treatment.

**Figure S17.** Relative expression levels of IAP in cancer cells after 13 days of treatment with free DOX, DOXIL or Aposomes, confirmed as western blot analysis.


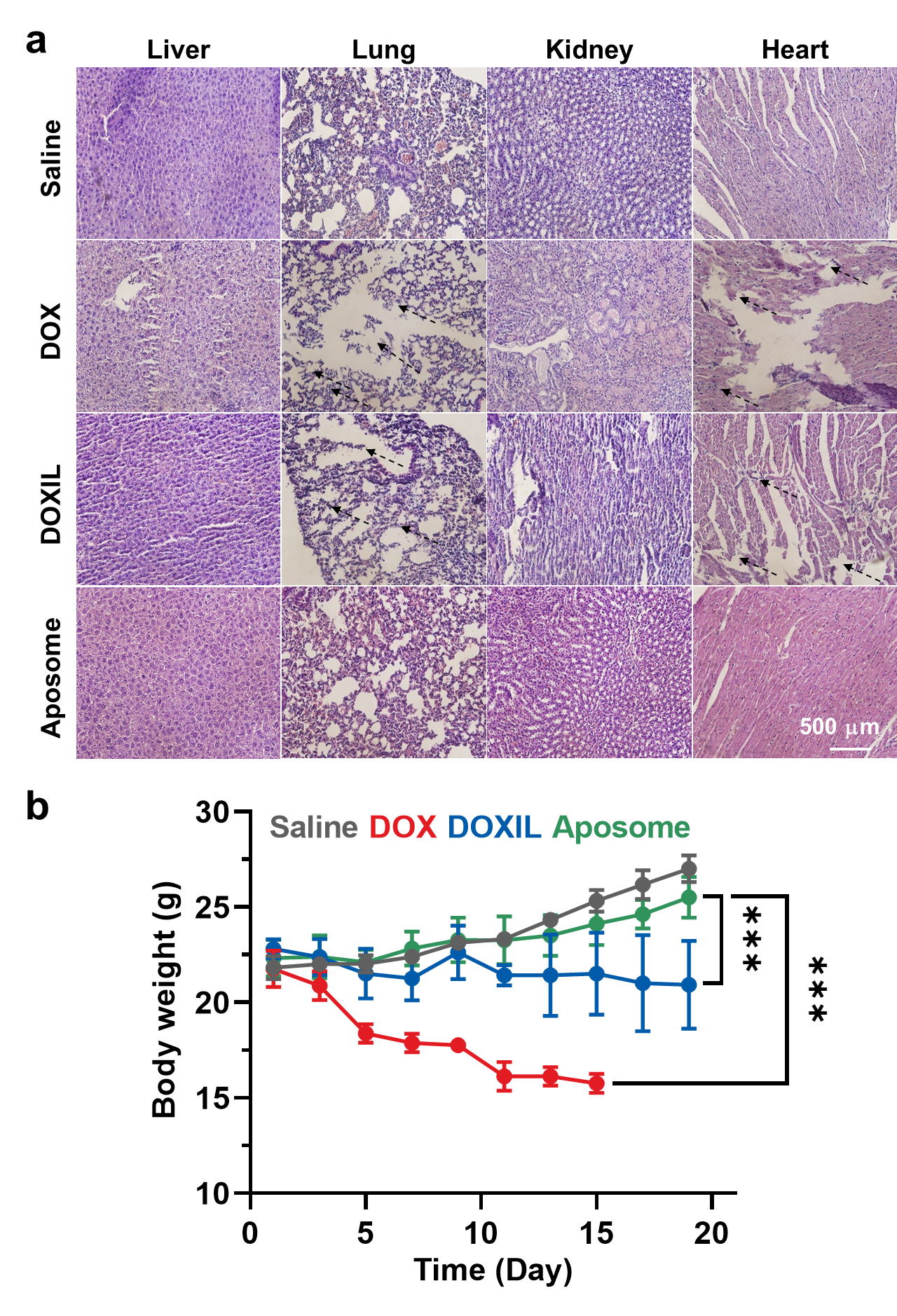


**Figure S18.** **(a)** Major organs (liver, lung, kidney and heart) stained with H&E on day 13 after treatment. **(b)** Body weight changes of mice during treatment with an equivalent 3 mg/kg DOX dose of free DOX, DOXIL or Aposomes once every three days.


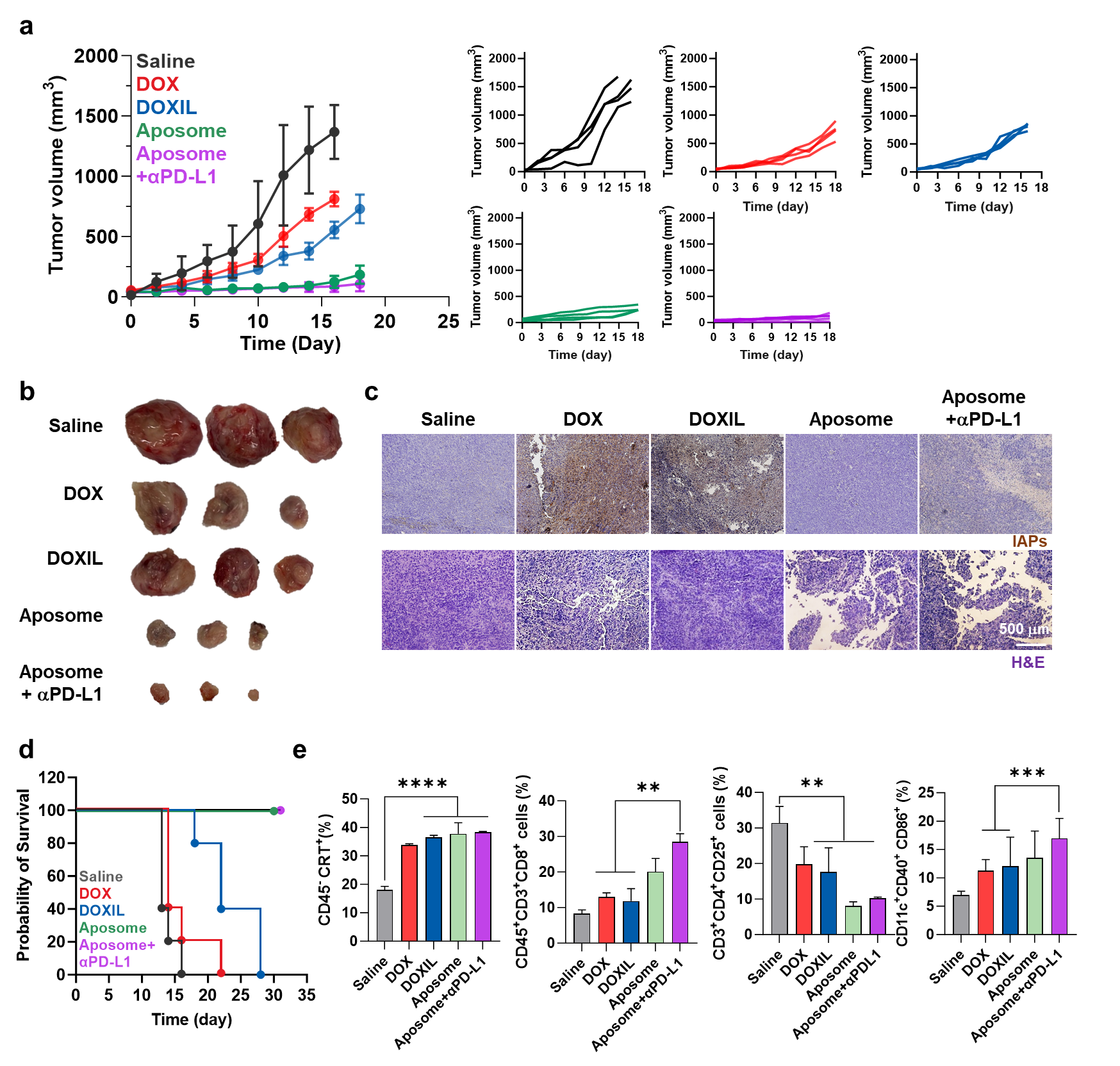


**Figure S19.** **(a)** Individual tumor growth curves of 4T1 breast tumor-bearing mice during treatment with an equivalent 3 mg/kg DOX dose of free DOX, DOXIL, Aposomes or Aposomes plus αPD-L1 Ab once every three days. **(b)** Optical images of tumor tissues on day 13 after treatment. **(c)** Tumor tissues stained with anti-IAPs antibody or TUNEL on day 13 after treatment. **(d)** Mice survival during treatment. **(e)** Various immune cells population within tumor tissues on day 13 after treatment.
